# Evaluation and integration of functional annotation pipelines for newly sequenced organisms: the potato genome as a test case

**DOI:** 10.1186/s12870-014-0329-9

**Published:** 2014-12-05

**Authors:** David Amar, Itziar Frades, Agnieszka Danek, Tatyana Goldberg, Sanjeev K Sharma, Pete E Hedley, Estelle Proux-Wera, Erik Andreasson, Ron Shamir, Oren Tzfadia, Erik Alexandersson

**Affiliations:** Blavatnik School of Computer Science, Tel Aviv University, Tel Aviv, Israel; Deptartment of Plant Protection Biology, Swedish University of Agricultural Sciences, Alnarp, Sweden; Institute of Informatics, Silesian University of Technology, Akademicka 2A, 44-100 Gliwice, Poland; Department for Bioinformatics and Computational Biology, Technical University of Munich, Arcisstraße 21, 80333 Munich, Germany; Cell and Molecular Sciences, The James Hutton Institute, Aberdeen, Scotland UK; Current affiliation: SciLifeLab, Stockholm University, Universitetsvägen 10, 114 18 Stockholm, Sweden; Department of Plant Science, The Weizmann Institute of Science, Rehovot, Israel

**Keywords:** Functional annotation, Gene ontology, Gene co-expression, Potato, Genomics

## Abstract

**Background:**

For most organisms, even if their genome sequence is available, little functional information about individual genes or proteins exists. Several annotation pipelines have been developed for functional analysis based on sequence, ‘omics’, and literature data. However, researchers encounter little guidance on how well they perform. Here, we used the recently sequenced potato genome as a case study. The potato genome was selected since its genome is newly sequenced and it is a non-model plant even if there is relatively ample information on individual potato genes, and multiple gene expression profiles are available.

**Results:**

We show that the automatic gene annotations of potato have low accuracy when compared to a “gold standard” based on experimentally validated potato genes. Furthermore, we evaluate six state-of-the-art annotation pipelines and show that their predictions are markedly dissimilar (Jaccard similarity coefficient of 0.27 between pipelines on average). To overcome this discrepancy, we introduce a simple GO structure-based algorithm that reconciles the predictions of the different pipelines. We show that the integrated annotation covers more genes, increases by over 50% the number of highly co-expressed GO processes, and obtains much higher agreement with the gold standard.

**Conclusions:**

We find that different annotation pipelines produce different results, and show how to integrate them into a unified annotation that is of higher quality than each single pipeline. We offer an improved functional annotation of both PGSC and ITAG potato gene models, as well as tools that can be applied to additional pipelines and improve annotation in other organisms. This will greatly aid future functional analysis of ‘-omics’ datasets from potato and other organisms with newly sequenced genomes. The new potato annotations are available with this paper.

**Electronic supplementary material:**

The online version of this article (doi:10.1186/s12870-014-0329-9) contains supplementary material, which is available to authorized users.

## Background

Potato (*Solanum tuberosum)* is the 3rd largest food crop in terms of human consumption [1]. It is therefore important for our food security, and understanding its genome is called for. Examples of major challenges in potato research are its sensitivity to drought stress and its lack of resistance to certain diseases, e.g., the oomycete *Phytopthora infestans,* which caused the Irish famine in the 1840’s. Farmers need to use large amounts of fungicides to protect their potato crops, thereby increasing the cost of cultivation and threatening the environment. For example, the global cost of protection and yield loss due to *P. infestans* has been estimated at €4800 M annually [2].

Recently, the potato genome (*Solanum tuberosum* group Phureja) was sequenced by the Potato Genome Sequencing Consortium (PGSC). The PGSC analysis of the genome reported gene models for 39,031 representative transcripts, and 56,218 including splicing variants [3]. In a later effort, the International Tomato Annotation Group (ITAG) produced new gene models by jointly analyzing the tomato and potato genomes [4]. These new gene models covered 34,727 and 35,004 predicted protein-coding genes for the tomato and the potato genomes, respectively. Unfortunately, few experimentally validated genes (e.g., by fluorescent-tagged proteins, or gene knock-outs) are available in newly sequenced genomes in which, unlike established model organisms, few genes have verified functions such as the case is for potato. Comprehensive and accurate functional annotation of the genes in such recently sequenced genomes is a prerequisite to efficient exploitation of these genomic data.

A key tool for functional annotation is the Gene Ontology (GO), which provides a structured set of defined terms representing gene properties [5]. The structure of gene ontology is composed of three major domains: *cellular component* (CC), the parts of a cell or its extracellular environment; *molecular function* (MF), the elemental activities of a gene product at the molecular level; and *biological process* (BP), which describes a set of functionally related molecular events. Thus, the complete GO structure provides a unified vocabulary of biological terms, which can also be used to evaluate biological similarity of different terms [6]. Annotating a gene means placing it within some or all of the three gene ontology domains.

Recent advances in plant science are marked by the rapidly increasing availability and quality of high-throughput sequencing data. The most basic usage of these data is gene function prediction, wherein GO plays a pivotal part. There are several computational suites like EXPANDER [7], MapMan [8], Mercator [9] and AmiGO [10] that enable biologists to run GO enrichment analyses in several plant model systems. This is usually done by first identifying a group of genes that behave similarly in a given expression dataset, seeking ontology terms highly enriched in the group, and associating the highly enriched functions with unannotated genes that belong to the same group. This process is sometimes called “guilt by association”. Automated gene function annotation is also relevant for well-investigated plant model organisms, such as *Arabidopsis thaliana,* tomato, *Brachypodium* and rice, wherein ~40% of the genes still do not have any known function [11].

In order to assign functional annotation to sequenced plant transcripts, researchers can use several sequence-based annotation pipelines. For a comprehensive summary of methods and principles behind automated functional annotation see [12]. Some recent efforts have been made to characterize the annotation quality of plant genomes. For example, Jaramillo-Garzón, et al. [13] used sequence features and showed high predictability of MF and CC terms and lower predictability of BP terms. However, the analysis was limited to a small subset of the GO terms (GO-Slim). Ramsak, et al. [8] presented GOMapMan, a tool for visualization and analysis of gene annotation in plants. In potato, information from orthologous gene families across 26 sequenced plant genomes was analyzed in order to increase the number of potato genes associated with GO terms [14]. Still, a robust, automated approach to evaluate and compare genome-wide annotation pipelines is direly needed.

**Figure 1:**
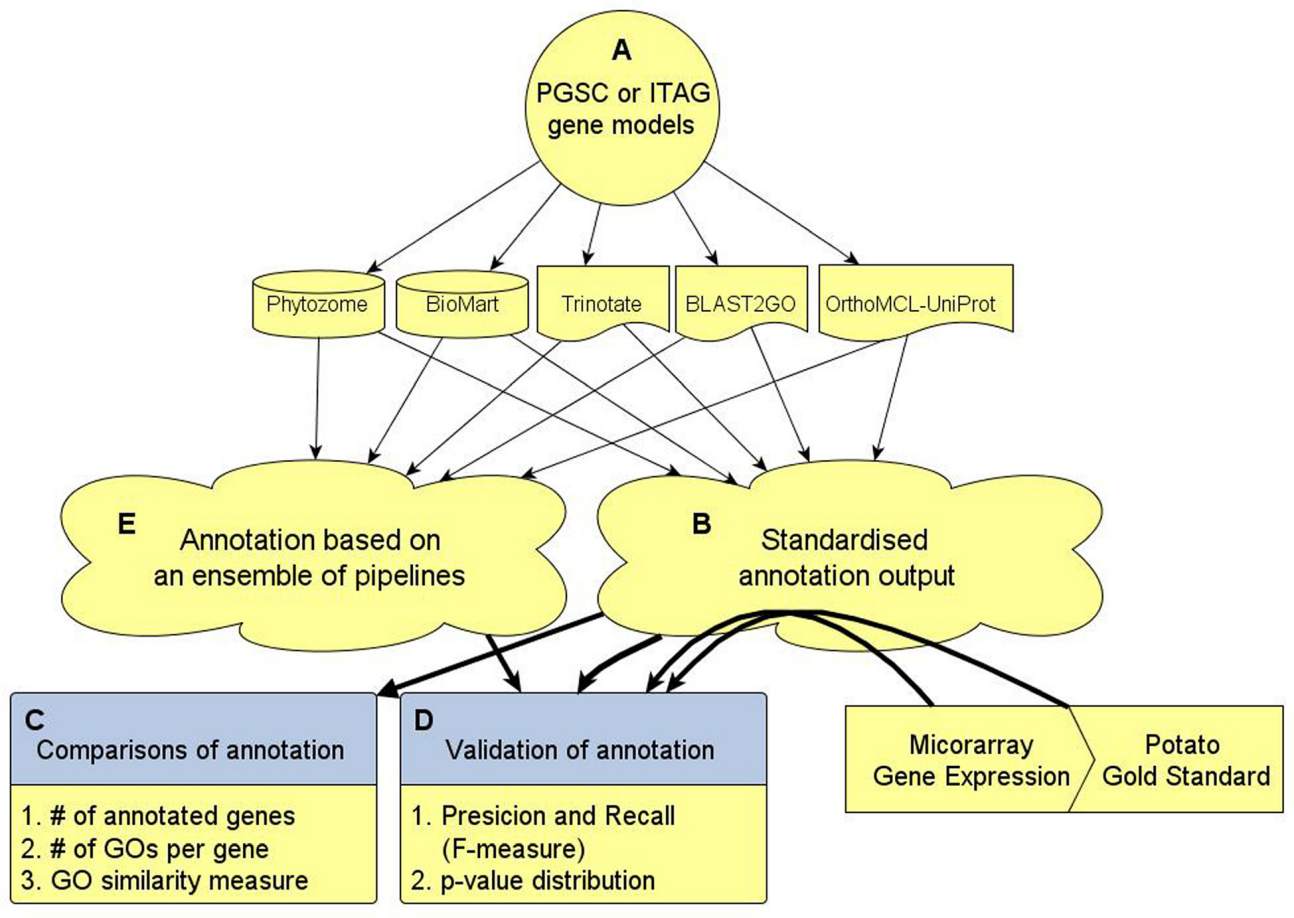
**Overview of pipeline comparison, validation of accuracy and integration processes. (A)** The PGSC and ITAG gene models were used as input for the six pipelines assessed. **(B)** The annotation from each pipeline was transformed into gene ID – GO term associations. **(C)** Annotations were compared by the number of annotated gene models, the number of GO terms associated per gene model, and GO similarity. **(D)** The quality and comprehensiveness of the annotation of each pipeline were calculated by comparing their predictions to experimentally validated annotation (gold standard). In addition, gene co-expression data were used to test if genes predicted to share the same GO processes are significantly co-expressed. **(E)** An integrated annotation using the ensemble of results of all pipelines was created and validated using the same criteria in D. Results of the ensemble annotations were compared to those of the individual pipelines.

A typical genome-wide functional annotation of newly sequenced organisms starts by using a single ‘default’ pipeline. Here, we analyzed the two sets of potato gene models, from the ITAG and PGSC. We compared six annotation pipelines: Trinotate HMM, Trinotate BLAST [15], OrthoMCL-UniProt [16], BLAST2GO [17], Phytozome [18] and InterPro2GO provided in BioMart [19] (Figure 1). These pipelines were chosen because they seek to provide a comprehensive annotation of the whole genome. Some of these pipelines are based solely on sequence similarity (BLAST), others rely on specific domains and some are based on clustering of groups of orthologous gene families. As we shall show, one clear conclusion of this work is that functional annotations of genomes should rely on more than one annotation pipeline.

By examining the GO terms generated by these pipelines, we demonstrate that they predict very dissimilar annotations (e.g., on average, less than 30% of the genes annotated by two pipelines are assigned with the same function). To evaluate the performance of the pipelines we first created a set of potato genes (hereafter referred to as “gold standard”), with known functional characterization, including genes from the well characterized biosynthetic Carotenoids pathway. We show that pipelines may have rather low accuracy compared to the gold standard. Since the size of the gold standard is rather modest (116 PGSC genes ids), we used an additional validation scheme based on gene expression data. Under the premise that genes participating in the same biological process should have more similar expression pattern than expected by chance, we evaluated the predictions of each pipeline based on its intra-process gene co-expression level. We show that while all pipelines provide much higher intra-process co-expression than expected by chance, there are large differences among the methods. We introduce a simple method to combine the results of the different pipelines into a single integrated annotation. Compared to the single pipelines, it improved gene coverage, prediction precision, and the overall co-expression of predicted GO processes. In addition to improved annotation of potato genes, our analysis provides generic tools that can be applied to improve the annotation of other newly sequenced plants.

## Results and discussion

### A compendium of the state-of-the-art annotation tools

In this study, we tested automatic annotation pipelines on the potato genome. We used six state-of-the-art tools for GO gene function prediction: (1) Trinotate HMM, (2) Trinotate BLAST [15], (3) OrthoMCL-UniProt [16], (4) BLAST2GO [17], (5) Phytozome [18], and (6) InterPro2GO [19]. See Methods and Additional file 1: Methods S1-4 for details. We note that every program has its own set of parameters and fitting the best parameter combination for a particular dataset is a substantial effort. The common practice in this area is to use published tools with the default parameter values (see e.g. [20,21]. If necessary, we then mapped its predicted functions to GO terms using automated mapping files such as Pfam2GO, and the genes and transcripts to protein identifiers. Thus, in our analysis a gene corresponds to either a transcript or a protein that appeared in the output of the pipelines. Next, the output of each pipeline was summarized as a set of predicted gene-GO term pairs. For each gene we then retained only the most “specific” GO terms. That is, in case a gene is associated with two GO terms A and B, but B is a generalization of A (i.e. an ancestor of A in the GO hierarchy), we excluded B. We call this step *ancestor removal*. Note that after filtering, many genes were still associated with more than one GO term, since a gene can have several associated annotations none of which is an ancestor of another. For the output of all pipelines, see Additional file 2: Table S1, Additional file 3: Table S2, Additional file 4: Table S3, Additional file 5: Table S4, Additional file 6: Table S5 and Additional file 7: Table S6 for PGSC, and Additional file 8: Table S7, Additional file 9: Table S8, Additional file 10: Table S9, Additional file 11: Table S10, Additional file 12: Table S11 and Additional file 13: Table S12 for ITAG. Although Gene Ontology has its limitations as it is biased towards what is already known, it is still a universal key tool for functional annotation inferring functionality based on sequence identity, domains and structure, and literature studies.

### Disparity among pipelines

The output from each pipeline can be represented as a triplet (P, G, GO) where P is the set of all predicted gene-GO term pairs (after ancestor removal), G is the set of genes covered by P, and GO is the set of GO terms covered by P. We measured the pairwise similarity between the triplets obtained from the six pipelines used in the study. Three different ways were used to compare the output of two pipelines A = (P_A_, G_A_, GO_A_) and B = (P_B_, G_B_, GO_B_). First, we measured the overlap between the predictions of the pipelines P_A_ and P_B_. This was done by calculating the ratio between the size of the intersection of P_A_ and P_B_ and the size of the union of P_A_ and P_B_. This measure is called the *Jaccard* score [22,23]. Second, we measured the similarity between the covered gene sets G_A_ and G_B_ of the pipelines by calculating their Jaccard scores. These two scores are complementary: the first measures the overall similarity between A and B, whereas the second measures the tendency of A and B to cover the same genes. However, these scores ignore the GO structure and thus they are oblivious to the functional similarity among different GO terms. Therefore, we also used a similarity score based on the semantic similarity of GO terms [24]. Given a specific GO type GT (BP or MF), for each gene we measured the semantic similarity between its GO terms in A and its GO terms in B. We then took the average over all genes as the similarity of A and B in GT (see Methods for details). As this score uses the structure of the GO hierarchy, we call it *structure-based*.

**Figure 2:**
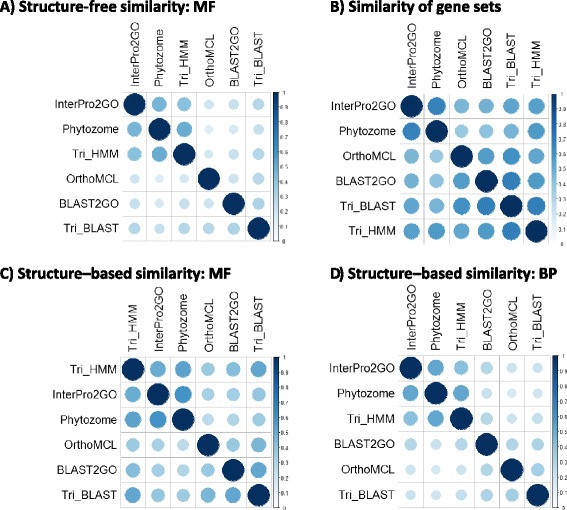
**Comparison of annotations of the PGSC genes by different pipelines.** Each similarity matrix shows all pairwise similarities between the pipelines. **(A)** Structure-free Jaccard similarity of the MF predictions of the pipelines. **(B)** Jaccard similarity of the gene sets covered by each pipeline. **(C)** Structure-based similarity between the GO MF predictions of the pipelines. Unlike **(A)**, the calculation here used the GO hierarchy to quantify the similarity of the predictions (see Methods). **(D)** Structure-based similarity between the GO BP predictions of the pipelines.

An example of the structure-free similarity of the predictions is shown in Figure 2A. The figure shows the pairwise Jaccard score between the PGSC MF predictions of the pipelines. Overall the similarity is low, averaging 0.27. Nevertheless, local patterns can be observed. For example, InterPro2GO, Trinotate HMM, and Phytozome were more similar (average 0.46). Figure 2B shows the Jaccard similarity between the PGSC genes annotated by the different pipelines. The mean similarity was a higher 0.54, which is still quite low. This indicates that different pipelines tend to cover different genes and, even when covering the same genes, they often associate distinct annotations to them. Even when re-computing the structure-free similarity restricted only for the genes shared by each pair of pipelines (considering both MF and BP predictions), the average score was only 0.27.

The structure-based MF and BP similarity of PGSC genes is summarized in Figure 2C and 2D. Similar matrices on ITAG data are shown in Additional file 1: Figure S1. Again, pipelines tend to be very different, with average similarity of 0.29 in BP and 0.42 in MF. The scores are higher than for the structure-free approach because the structure-based approach assigns higher scores when predictions are different but biologically similar. Also, like in the structure-free scores in Figure 2A, InterPro2GO, Trinotate HMM, and Phytozome formed a cluster both in BP and in MF. Taken together, the discrepancies among pipelines show that pipelines differ in the sets of genes they cover, and the annotation of the same genes in different pipelines can be quite dissimilar.

### Ensemble of pipelines

**Figure 3:**
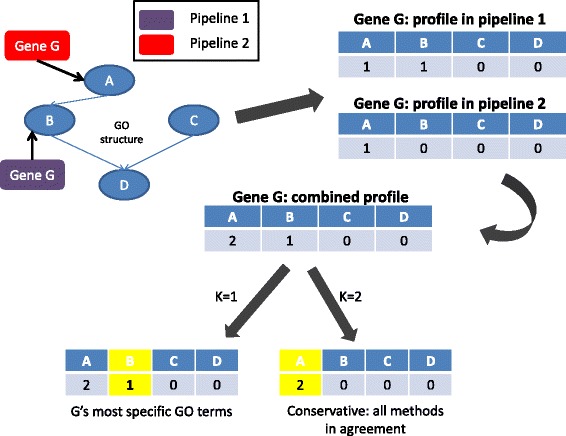
**A simple example of the ensemble algorithm.** The input (top left) is a set of GO terms, the GO graph, and association between genes and GO terms. The example shows the ensemble process of a single gene G. First, the *pipeline-specific* gene profiles are calculated (top right). A GO term is assigned a value ‘1’ in the profile if G is associated with it or with at least one of its descendants and ‘0’ otherwise. Second, the combined profile of G is the sum of its pipeline-specific profiles. The scores in the combined profile show how many pipelines agree with each of G’s GO term association. Given a threshold k, the GO terms with a combined score lower than k are removed to provide a final list of GO terms associated with G (bottom). Each different value of k constitutes a different variant of the algorithm.

The marked disparity in gene annotation by different pipelines calls for an integration of the different predictions in order to provide a unified potato gene annotation. We developed a simple ensemble algorithm inspired by previous studies [25]. Our algorithm takes as input the predictions of all pipelines and for each gene merges its predictions into a vector of scores denoted as the gene’s *combined profile* (Figure 3). Briefly, we first calculate the *pipeline-specific* gene profiles. For a specific pipeline that predicted the pair (G, t), where G is a gene and t is a GO term, the t-th position of the profile is 1 if G is associated with t or at least one of its descendants, and otherwise it is 0 (top right in Figure 3). The combined profile of each gene G is the sum of its pipeline-specific profiles (Figure 3 right). The value in the combined profile of a gene shows how many pipelines agree with each gene-GO term association. Given a threshold k, for each gene we report all GO terms with a combined score ≥ k. This process produces a list of GO terms for each gene. We call this variant *Ensemble-k.* Finally, we apply the ancestor removal filter described above. Thus, each value of k produces a different variant of the ensemble algorithm. Figure 3 shows a toy example of Ensemble-1 and 2. For clarity, in the next sections we use the name *annotation method* for both pipelines and variants of the ensemble algorithm. We also tested a more involved supervised ensemble method, which in addition ranks the pipelines by their average F-measure against a gold standard (see below), but this did not improve the results (see Additional file 1: Method S6).

**Figure 4:**
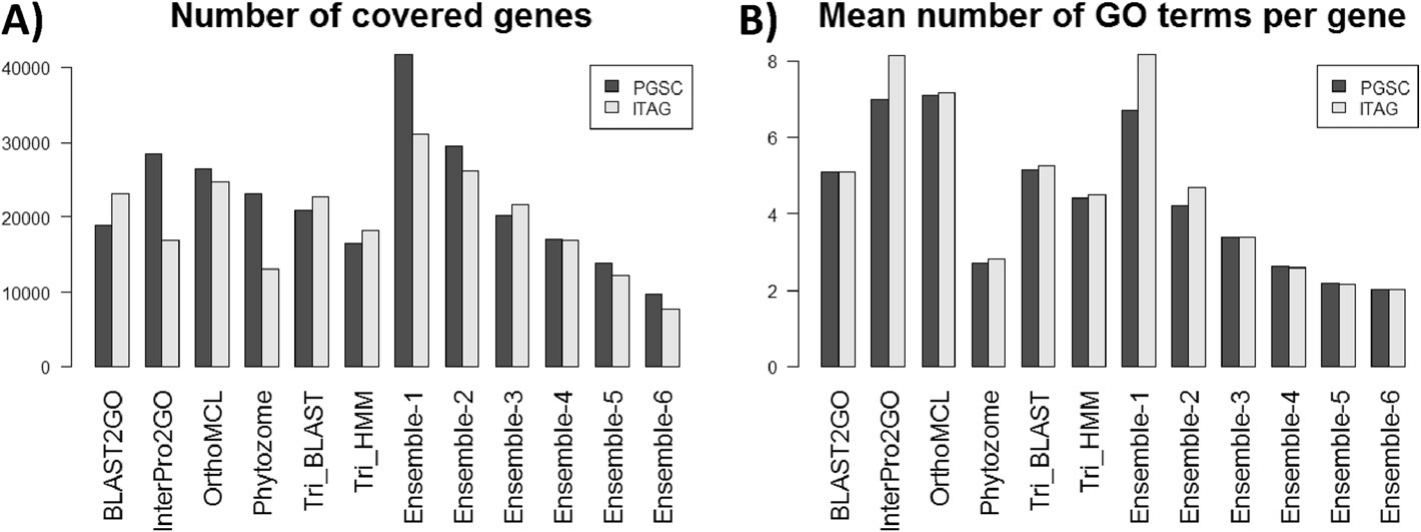
**Gene coverage and mean number of GO terms per gene (NGPG).** For each annotation method (i.e., a pipeline and a variant of the ensemble algorithm) the gene coverage **(A)** and NGPG **(B)** are shown both for PGSC and ITAG gene models.

We compared the annotation methods in terms of gene coverage and the average *n*umber of GO terms *p*er *g*ene, which we denote as NGPG. Ideally, gene coverage should be as high as possible, while NGPG should be low [26]. The results are shown in Figure 4A and 4B. One can observe marked differences between the different pipelines, and between ITAG and PGSC gene models. For example, based on PGSC data, InterPro2GO and OrthoMCL-UniProt have the highest gene coverage (29,445 and 26,371, respectively), and NGPG score (7 and 7.1, respectively). However, based on ITAG data, OrthoMCL-UniProt’s results were similar to those for PGSC, while for InterPro2GO the number of genes dropped under 20,000 and the NGPG score increased to 8.1 (Figure 4B).

Figure 4A and 4B also show the gene coverage and the NGPG of the ensemble algorithm. As expected, using either Ensemble-1 or 2 increased the gene coverage compared to the single pipelines using both ITAG and PGSC gene models. For example, based on PGSC the number of covered gene models (including splicing variants) was 41,668 (k = 1) and 29,495 (k = 2). Larger k values led to a sharp decrease in gene coverage, such that even single pipelines covered more genes. Using Ensemble-1, the NGPG score was similar to the highest score obtained by a single pipeline, reaching a score of 6.70 on PGSC data, and 8.15 on ITAG data. Ensemble-2 led to a sharp decrease in NGPG: 4.39 on PGSC, and 4.68 on ITAG.

In summary, our results show that the ensemble algorithm increases the gene coverage considerably without increasing the NGPG score. Ensemble-1 increased gene coverage by more than 5000 genes on both ITAG and PGSC data, while keeping the NGPG score similar to that of the highest single pipelines. Ensemble-2 increased the gene coverage only moderately compared to the single pipelines but the NGPG score declined sharply compared to all pipelines (except Phytozome, but the latter has low gene coverage), hence providing much more focused annotations. In the next sections we demonstrate that the aforementioned improvements were not achieved at the expense of precision.

### Validation using the potato gold standard

To evaluate predictions of the different annotation methods we compiled a gold standard of 838 and 724 gene-GO term pairs based on PGSC and ITAG data, respectively, using manual annotation by experts (see Methods and Additional file 14: Table S13, Additional file 15: Table S14 and Additional file 16: Table S15). The number of genes included in the gold standard (43 with literature references, which are mapped to 116 PGSC gene ids, see Additional file 14: Table S13), is small, but in an organism such as potato it still contains the majority of genes with experimental evidence. We evaluated the annotation methods by calculating their GO-based precision and recall. Use of the GO structure to calculate scores for gold standard validation has been previously suggested by [27]. The GO-based recall of a gene measures the extent to which its terms according to the gold standard are covered by its predicted GO terms. The GO-based precision of a gene measures the extent its predicted GO terms match the gold standard terms. For each pipeline we calculated the average precision and average recall (over the genes) and report the F-measure, which is the harmonic mean of the precision and the recall [28]. See Methods for a full description of these calculations.

**Figure 5:**
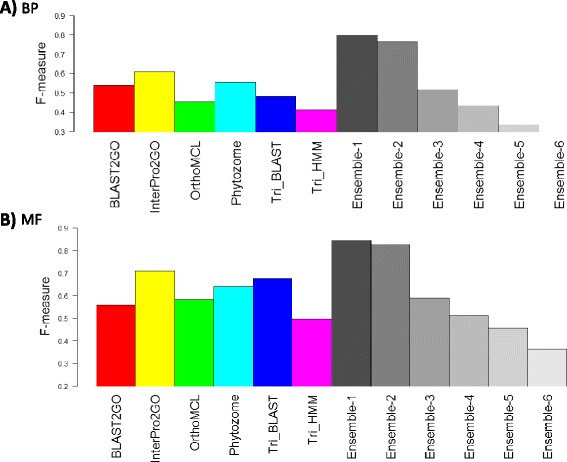
**Validation of annotations based on gold standard.** For each annotation method (i.e., a pipeline and a variant of the ensemble algorithm) the F-measure of the gold standard validation is shown on PGSC gene models, see Methods for a full description of the scores. A score of 1 means perfect agreement between an annotation method and the gold standard. A score close to zero means poor concordance with the gold standard. **(A)** F-measure of the BP annotations. **(B)** F-measure of the MF annotations. The results show that both in BP and MF the ensemble algorithm improves the results considerably when used with k is 1 or 2.

The results of the validation based on PGSC and ITAG data are illustrated in Figures 5 and Additional file 1: Figure S2, respectively. Figure 5A shows the F-measure for BP GO terms. Ensemble-1 and 2 reached F-measures of 0.8 and 0.77, respectively, while the top performing pipeline was InterPro2GO with only 0.61. Figure 5B shows the F-measure on the MF gold standard. Ensemble-1 and 2 reached F-measures of 0.84 and 0.83, respectively, whereas the top performing pipeline was InterPro2GO with an F-measure of only 0.71. Thus, the results are in agreement with the BP validation: Ensemble-1 and 2 performed best and improved upon the single pipelines. Taken together, our results indicate that Ensemble-1 and 2 provide a significant improvement in comparison to single pipelines.

### Validation using gene expression data

An obvious disadvantage of any gold standard is that it is limited to experimentally validated genes and subject to the opinion of experts. Consequently, we added an additional validation based on gene co-expression analysis, where we measured the ability of pipelines to predict the same GO-term to highly co-expressed genes. Our co-expression analysis is based on the gene expression of 12,956 genes in 326 expression profiles from over 20 microarray studies. We used the Pearson correlation coefficient to measure co-expression between genes.

We used the gene pairwise co-expression scores to validate predicted GO BP terms. In order to reduce noise, we ignored terms with >500 genes, or with fewer than five genes. Given a set of genes predicted to be associated with the same GO term according to a specific annotation method, we tested if the level of co-expression among its genes is higher than expected by chance (see Methods for details). Thus, for each term in a specific annotation method we calculated a single p-value. To summarize these values when comparing methods we calculated two scores: (1) the number of GO terms with p <0.001, and (2) the percentage of GO terms with p <0.001 (out of all predicted terms with at least three genes). The former is a measure of coverage of significant GO terms, whereas the latter is a measure of quality of the predicted GO BP terms. Similarly to the gold standard, this analysis simply aimed to compare pipelines. Future work can use similar approaches to select highly co-expressed GO terms from different pipelines for subsequent analyses.

**Figure 6:**
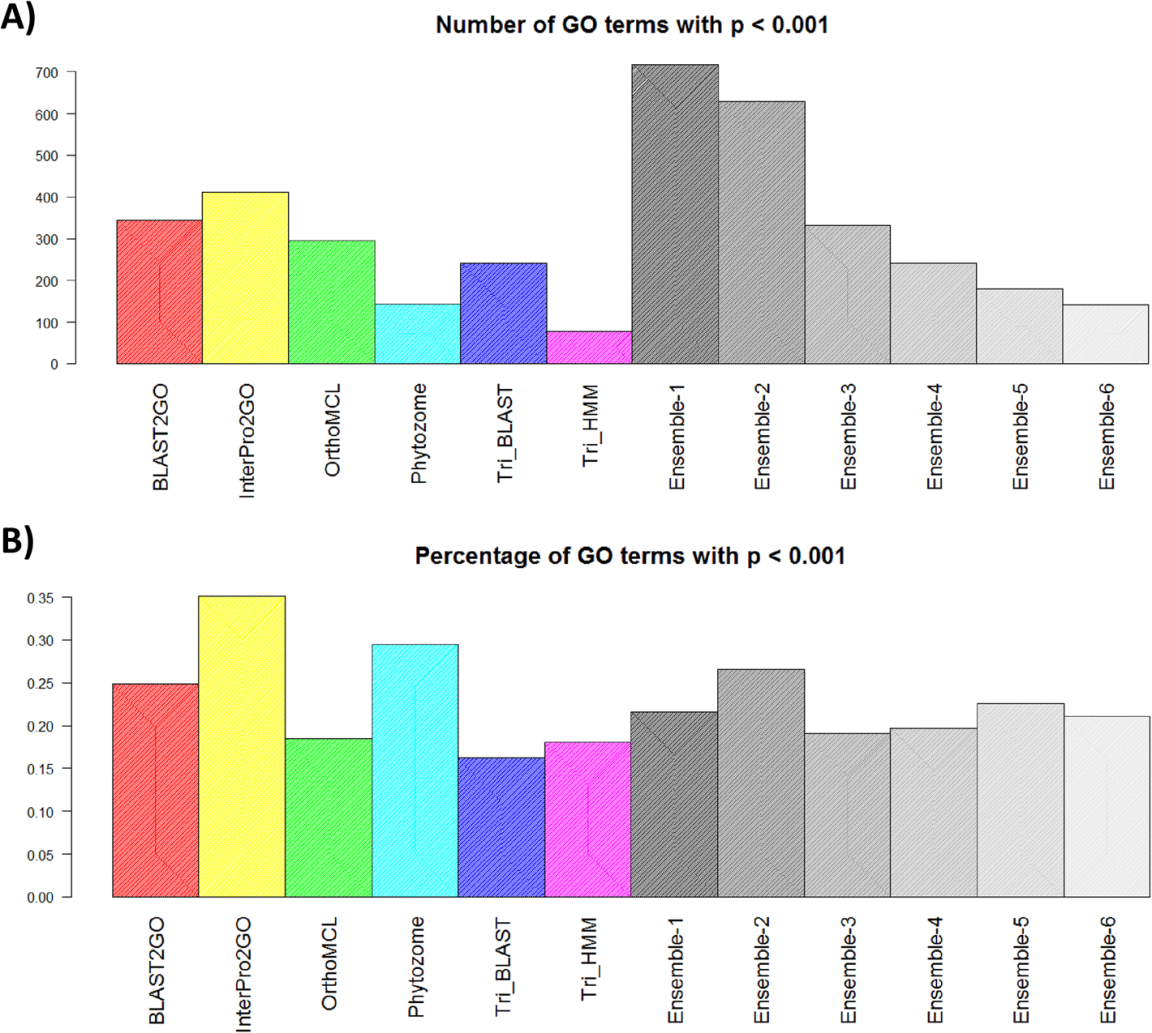
**Validation of annotations based on co-expression.** Given a set of PGSC genes linked to a biological process by a specific annotation method (i.e., the pipelines or a variant of the ensemble algorithm) the average co-expression of the genes was compared to that of random gene sets. For each annotation method the number of GO terms with p <0.001 **(A)**, and the percentage of GO terms with p <0.001 **(B)** are shown. Ensemble-2 has a lower percentage of significant GO terms compared to the best single pipeline (BioMart), but it has >1.5 fold more significant GO terms.

The results of the gene co-expression validation based on PGSC data are shown in Figure 6. See Additional file 1: Figure S3 for results of ITAG. The top two pipelines in terms of the number of significant GO terms were InterPro2GO (n = 411) and BLAST2GO (n = 345). The top two pipelines in terms of the percentage of significant GO terms were InterPro2GO (35%) and Phytozome (30%). The ensemble algorithm markedly improved the number of significant GO terms: Ensemble-1 achieved 718, and Ensemble-2 achieved 650. However, the ensemble methods did not improve upon the single pipelines in terms of the percentage of significant GO terms: Ensemble-1 and 2 achieved 22% and 27%, respectively. Nevertheless, the score of Ensemble-2 was better than all pipelines except for InterPro2GO and Phytozome. Thus, the ensemble approach provided an improvement of at least 1.5-fold in the number of significant GO terms, at the expense of a drop of 8% in the percentage of significant GO terms compared to the best pipeline. Note that the co-expression and the GO analyses are complementary, since the gold standard genes do not manifest unusually high co-expression (see Additional file 1: Methods S7).

### Merging the different merits using a rank-based comparison

Our analysis shows that the ensemble approach is beneficial according to most criteria. However, since we used multiple ways to score the methods, it is hard to decide which k value is best and which pipelines are better. To provide a clear unified view we used a non-parametric rank-based consolidation of the different scores [29]. In the previous sections, for each annotation method we calculated two F-measure scores in the gold standard analysis and two scores in the gene co-expression analysis. In addition, we compared the annotation methods by their gene coverage and NGPG. Note that when ranking methods by their NGPG score, lower scores are better. In contrast, when ranking methods by their gene coverage, higher scores are better. To consolidate these different scores, we used six rankings: by gene coverage and the NGPG score, by the two F-measures of the gold standard validation and by the two scores of the gene co-expression validation. We reversed the scores when necessary so that rank 1 was the best for each method, averaged the rankings and ranked the methods by their average rank. We call this score *rank-merge.*

**Figure 7:**
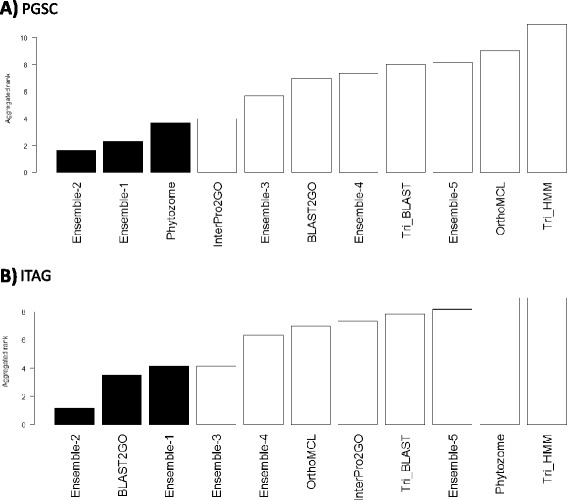
**Rank-based consolidation of the different figures of merit.** A non-parametric rank-based consolidation of the different scores of the annotation methods was used for a unified comparison. First, six rankings were calculated: by gene coverage, by NGPG, by the two F-measures of the gold standard validation, and by the two gene co-expression validations scores (i.e., the number and the percent of significant GO terms). To merge the different rankings we used the average rank. The results show that both for PGSC (panel **A**) and for ITAG (panel **B**), Ensemble-2 has the best average rank.

Figure 7 displays the rank-merge results on PGSC (A) and ITAG (B) data. The top three methods are colored black. In both cases the top method was Ensemble-2, with an average rank of 1.66 in PGSC and 1.16 in ITAG. Among the different pipelines evaluated, Phytozome obtained the top score for PGSC data with an average rank of 3.66 while BLAST2GO obtained top score for ITAG data with an average rank of 3.50. Note that Ensemble-1, 2, and 3 were ranked consistently high in both tests. See also Additional file 17: Table S16 for PGSC and Additional file 18: Table S17 for ITAG. Thus, we conclude that the ensemble approach, especially with k = 2, is beneficial and can assist in integration of different gene function prediction pipelines. See Additional file 1: Method S5 for details on reproducing the results and applying the pipeline to new genomes.

Note that using k = 1 is equivalent to assigning to each gene all its annotations from all pipelines (and their ancestors) and then performing ancestor removal. While this method is the most intuitive ensemble, we show here that varying the k parameter can improve the annotation of genomes.

A seemingly natural test case for our approach is to evaluate it in predicting function of Arabidopsis genes. However, it is not clear how this can be done in a rigorous and unbiased manner. Tools for functional annotation of genes in newly sequenced plants are heavily dependent on sequence similarity to genes in model species such as Arabidopsis. In order to test such tools in predicting Arabidopsis gene functions, one has to exclude all the annotations directly – or indirectly – derived from Arabidopsis. Doing so would entail tracing indirect annotation sources, which often are not recorded in the pipelines. Instead, we used the newly sequenced potato genome along with experimentally verified gene functions and rich gene expression data in our evaluation.

## Conclusion

For recently sequenced, non-model organisms, automatic functional annotation of genes, which also mainly relies on sequence-based prediction, often suffers from low gene coverage and poor specificity. We confirmed that this is the case for the potato genome by analyzing six state of the art annotation pipelines.

We observed that the predictions of different pipelines for functional annotations of genes are markedly different, in spite of the fact that all pipelines are based on sequence analysis. We showed that combining predictions from several pipelines increases both the coverage and the accuracy of gene ontology predictions. The simple ensemble approach used here could be applied easily to other sequenced genomes and improve functional annotation by taking advantage of different GO prediction tools. However, a comparison of the consistency among pipelines is not enough when the goal is to either select the best pipeline or to integrate the different predictions. The pipelines should also be evaluated based on the precision of their predictions. The most intuitive way is to compare the pipelines to a set of known annotations. However, in newly sequenced organisms such as potato, known annotations are scarce in the main public databases. To overcome this, we compiled a gold standard of experimentally-validated gene-GO associations. Although this gold standard is relatively small, we have found it useful for comparing pipelines. Furthermore, to overcome the limited number of genes in the gold standard, we used a second validation method based on gene co-expression testing the ability of pipelines to predict co-expression of genes associated to the same GO-term.

Finally, we introduced an integrated annotation of the different pipelines that outperformed the single pipelines both in the gold standard validation and in the co-expression validation. Our integration approach depends on selecting a parameter k that corresponds to the stringency by which we filter out gene-GO associations. That is, when associating a gene to a GO term, at least k pipelines must agree with this association. Thus, we have implicitly assumed that each of the pipelines we used has meaningful predictions. Moreover, all pipelines have the same weight in the integration process. Future analyses can seek methods that give more weight to better pipelines, or add an initial step that filters out pipelines of exceptionally low prediction quality. The new functional annotations of the potato genome as well as for the probes on the JHI Solanum tuberosum microarray are available with this paper (Additional file 17: Table S16, Additional file 18: Table S17 and Additional file 19: Table S18). We also provide tools as open source R code for implementing the methodology with additional pipelines and for other sequenced organisms.

## Methods

### Executing the functional annotation pipelines

We defined a *pipeline* as an automated process that predicts association between genes and functions. The input to a pipeline can be DNA sequence, protein sequence, or protein domains. The output of a pipeline is a set of pairs in the form of (gene ID, GO term ID). We ran all pipelines for the ITAG (potato.Sotub.proteins.itag.v1.fasta) and PGSC (PGSC_DM_v3.4_pep_representative.fasta) gene models separately, using default settings as follows:

#### The OrthoMCL-UniProt pipeline

We ran the OrthoMCL [16] pipeline in two steps:

1. Building the clusters of homologs: We retrieved from Phytozome (v9.1) 16 plant proteomes, covering the whole plant phylogeny. Together with the proteomes predicted from the potato PGSC and ITAG gene models, we aligned the proteomes against each other using blastp [30]; (parameters: −e-value: 1e-05 -outfmt 6). We then used OrthoMCL v2 to build clusters of homologous proteins.
2. Annotating GO terms: To annotate every protein sequence of the 18 complete plant proteomes with GO terms we ran a blast search against the entire UniProt database (version 2013_08) [31] with an e-value cut-off of 1e-10. For every protein sequence we kept a ranked list of the ten best hits (i.e. hits with the lowest e-value). We associated the first hit in the list that had GO annotation in UniProt. An OrthoMCL cluster then inherits all GO terms associated with its proteins, and each PGSC (and ITAG) protein inherits the GO terms of its cluster.

For complete protocol details refer to the Additional file 1: Method S2.

#### The BLAST2GO pipeline

Using the BLAST2GO interface [17], we blasted the PGSC and ITAG protein sequences against the NCBI NR database (blastp parameters: −e-value: 1e-05 -max_target_seqs 20 -outfmt 5). We then loaded the blastp output files into Blast2GO (v2.6.6, with default parameters) and assigned GO terms to the PGSC and ITAG sequences according to its output.

#### The trinotate pipeline

In the Trinotate suite [15] we used default settings for the NCBI-BLAST (SwissProt), HMMER [32], and Pfam [33]. For complete protocol details refer to the Additional file 1: Method S3.

#### The phytozome pipeline

We downloaded the potato annotation from Phytozome v9.1 [http://www.phytozome.net/potato.php; 18] (http://www.phytozome.net/potato.php). The gene annotation is *Solanum tuberosum* Group Phureja DM1-3 516R44 (CIP801092) *Genome Annotation v3.4 mapped to* pseudomolecule *sequence* (PGSC_DM_v3_2.1.10_pseudomolecules.fa).

#### InterPro2GO data from BioMart

We downloaded the potato data from (http://central.biomart.org/). GO terms in BioMart are derived from the semi-automated InterPro2GO [19].

#### Formatting pipelines

In order to compare pipelines, we mapped their predicted annotation to a set of common Gene Ontology (GO) terms. If the original pipeline output was not in GO term IDs it was mapped to GO IDs using the gene ontology consortium mapping files for GO terms. We applied this procedure to the pipelines Trinotate, InterPro2GO, BLAST2GO, Phytozome, and in mapping of orthologous and paralogous gene families in 18 sequenced plant species by OrthoMCL clustering.

### Composing the potato ‘gold standard’

A ‘gold standard’ set of potato genes was constructed based on literature evidence from functional gene studies by wet-lab experiments in potato reported in PlantCyc [http://pmn.plantcyc.org/PLANT/organism-summary] and a few additional studies on potato [34-37]. In total a list of 43 potato genes/proteins was created (Additional file 14: Table S13). These protein names were searched for their corresponding identifiers published by the PGSC [3], resulting in 116 unique PGSC gene identifiers.

The aforementioned list of genes matched 1658 GO terms from all six tested pipelines. Each gene-GO term association was then manually scored with the help of literature searches in an unbiased manner, where the experts assigning scores to GO-associations did not know from which pipeline the annotation originated. Every GO term in the set was scored as ‘1’ (low evidence), ‘2’ (neutral or unknown) and ‘3’ (high evidence). In the final analysis only association scores of 3 were used for the gold standard, producing 838 annotations (Additional file 15: Table S14). To perform analyses on both gene models, PGSC genes were mapped to ITAG genes using BLAST (identity >95%, length >100 amino acids). This produced an ITAG gold standard with 724 annotations (Additional file 16: Table S15).

### Comparing pipelines and gold-standard evaluation

#### Mathematical notations

In the Results section we sketched the calculations for comparing pipelines and evaluating pipelines against the gold standard. Here, we provide a full description of these calculations. For this purpose we start here with more detailed definitions.

Let G be the set of all genes in the tested organism and let T be the set of all GO terms. The output of a pipeline *P* is a set of pairs *P = {p*_*1*_*, …, p*_*k*_*}* where each *annotation pair* p_i_ = (g_i_,t_i_) is an association between a gene g_i_ (in G) and a GO term t_i_ (in GO). Let BP(P) be the subset of P resulted from taking all pairs in P in which the term t is a biological process. Similarly, define MF(P) for molecular function, and CC(P) for cellular component. Below we define functions of pipelines. Note that by definition each of BP(P), MF(P), and CC(P) is a set of pairs. Thus, in the definitions below P is either the original output of a pipeline or the result of applying BP, MF, or CC on it.

We define *Genes(P)* as the set of genes covered by P and *Terms(P)* as the set of GO terms covered by *P*. We define *Genes(P,t)* as the set of genes associated with a GO term *t* according to *P*, and *Terms(P,g)* as the set of GO terms associated with a gene *g* according to *P*. Finally, we denote *Sem(t*_*i*_*,t*_*j*_*)* as the semantic similarity between two GO terms *t*_*i*_ and *t*_*j*_. Semantic similarity here is a measure that quantifies the closeness of two terms in the GO graph. There are several ways to calculate semantic similarity among GO terms. In this study we used Wang’s method [6,24].

### Jaccard coefficient between two pipelines

The Jaccard coefficient is a generic measure of similarity between two sets. It is defined as the ratio between the size of the intersection of the sets and the size of the union of the sets. For example, given two pipelines *P*_*1*_ and *P*_*2*_, denote *intersect(P*_*1*_*,P*_*2*_*)* as the set of annotation pairs that are both in *P*_*1*_ and in *P*_*2*_, and let *union(P*_*1*_*,P*_*2*_*)* be the set of annotation pairs that are either in *P*_*1*_ or in *P*_*2*_. The Jaccard coefficient *J*_*pipeline*_*(P*_*1*_*,P*_*2*_*)* is the ratio between the number of annotation pairs in *intersect(P*_*1*_*,P*_*2*_*)* and the number of annotation pairs in *union(P*_*1*_*,P*_*2*_*)*. In addition, we calculate the Jaccard coefficient *J*_*Genes*_*(P*_*1*_*,P*_*2*_*)* between the gene sets *Genes(P*_*1*_*)* and *Genes(P*_*2*_*)* to measure the tendency of two pipelines to annotate the same genes.

### Structure-based similarity between two pipelines

The Jaccard measure above is oblivious to the functional similarity among GO terms. Thus, we used semantic similarity as a means to define a structure-based similarity between two pipelines *P*_*1*_ and *P*_*2*_. We start by defining the similarity between the set of annotations of a single gene. Given a gene g our goal is to measure the semantic similarity between *Terms(P*_*1*_*,g)* and *Terms(P*_*2*_*,g)*. As a first step we define the similarity between a single GO term *t* and a set of GO terms *T’* as:

$$ Sim\hbox{'}\left(t,{T}^{\hbox{'}}\right) = \underset{t\hbox{'}\in T\hbox{'}}{ \max }Sem\left(t,t\hbox{'}\right) $$

This score is high only if T’ contains *t* or similar GO terms. Next, we use this score to calculate the similarity between *Terms(P*_*1*_*,g)* and *Terms(P*_*2*_*,g)* using the running-max-average [6]:

$$ rmaxa\left({P}_1,{P}_2,g\right) = \frac{{\displaystyle {\sum}_{t_i\in Terms\left({P}_1,g\right)}}Sim\hbox{'}\left({t}_i,\  Terms\left({P}_2,g\right)\right) + {\displaystyle {\sum}_{t_j\in Terms\left({P}_2,g\right)}}Sim\hbox{'}\left({t}_j,\  Terms\left({P}_1,g\right)\right)}{\left| Terms\left({P}_2,g\right)\right|+\left| Terms\left({P}_1,g\right)\right|} $$

This score will be high only if *Terms(P*_*1*_*,g)* covers the biological functionalities of *Terms(P*_*2*_*,g)* and vice versa. Finally, the overall similarity between *P*_*1*_ and *P*_*2*_ is the average gene-wise similarity:

$$ Sim\left({P}_1,{P}_2\right)=\frac{{\displaystyle {\sum}_{g\in Genes(P)\cup Genes\left({P}_2\right)} rmaxa}\left({P}_1,{P}_2,g\right)}{\left| Genes(P)\cup Genes\left({P}_2\right)\right|} $$

### GO-based precision and recall

The calculations above measure similarity among pipelines. Here we define a way to measure the precision and recall of a pipeline *P* compared to a gold standard *GS*. Similarly to *P, GS* is a set of annotation pairs *{gs*_*1*_*, …, gs*_*k*_*}* where each pair *gs*_*i*_ *= (g*_*i*_*,t*_*i*_*)* is an association between a gene *g*_*i*_ (in *G*) and a GO term *t*_*i*_ (in *T*). We first define the precision of a single gene *g*. The GO-based *precision* of pipeline *P* for gene *g* measures the extent by which *Terms(P,g)* is covered by *Terms(GS,g)*:

$$ prec\left(P,GS,g\right) = \frac{{\displaystyle {\sum}_{t_i\in Terms\left(P,g\right)}}Sim\hbox{'}\left({t}_i,\  Terms\left(GS,g\right)\right)\ }{\left| Terms\left(P,g\right)\right|} $$

The *precision* of *P* is defined as the average precision of the genes in *Genes(G)*:

$$ prec\left(P,GS\right)=\frac{{\displaystyle {\sum}_{g\in Genes(GS)} prec\left(P,GS,g\right)}}{\left| Genes(GS)\right|} $$

The *GO-based recall* of pipeline *P* for gene *g* measures the extent by which *Terms(P,g)* covers *Terms(GS,g)*:

$$ recall\left(P,GS,g\right)=\frac{{\displaystyle {\sum}_{t_i\in Terms\left(GS,g\right)}Si{m}^{\prime}\left({t}_i, Terms\left(P,g\right)\right)}}{\left| Terms\left(GS,g\right)\right|} $$

The *recall of P* is defined as the average recall of the genes in Genes (G):

$$ recall\left(P,GS\right)=\frac{{\displaystyle {\sum}_{g\in Genes(GS)} recall\left(P,GS,g\right)}}{\left| Genes(GS)\right|} $$

### Microarray data preprocessing and normalization

We have integrated potato gene expression data from over 20 studies based on the Agilent JHI Solanum tuberosum 60 k v1 microarray (ArrayExpress ID: E-MTAB-1655) processed at the James Hutton Institute using standard Agilent recommended methodologies [38]. The studies included 326 conditions derived from the following treatments: moderate heat-stress [38], short- and long-day growth regimes [39], bruising, phosphorous growth regimes, acidity, *Phytopthora infestans* infection [40], and phosphite [41], BABA [14], ABA, brassinosteroid, SA treatment. Varietal differences and tuber, stem and leaf tissues were included.

We applied quantile normalization using the Limma package [42] and subtracted the background intensity from the foreground intensity for each spot using the ‘normexp’ method [43]. Our normalized expression matrix contained 52,998 probes. In order to reduce statistical noise and to focus on genes with high variation we removed both probes with consistently low expression values across the samples and probes with low variance. Thresholds for probe removal were adjusted as proposed in [44], see Additional file 1: Method S4 for more details. 14,000 probes remained in the data. These probes were mapped to 12,956 genes, approximately the same amount of genes analyzed in Tzfadia, et al. [44].

### Evaluating co-expression of predicted GO processes

Given a gene set U associated with a specific GO term, and a gene expression matrix X with genes as rows, we first calculate the Pearson correlation between all pair of genes in U using their expression profiles in X. To evaluate if the correlations in U tend to be higher than expected by chance we sample random gene pairs in X and calculate their correlation to get a distribution of random correlation scores. We used the Kolmogorov-Smirnov test to compare the real correlations scores of U to the random correlation scores. To improve robustness, we repeated this process 50 times for each gene set U and used the mean p-value over all repeats.

## Additional files

Additional file 1: Figure S1. ITAG pipeline similarity, **Figure S2.** ITAG gold standard validation, **Figure S3.** ITAG gene expression validation and **Methods S1-5**. Additional file 2: Table S1. InterPro2GO PGSC pipeline output. Additional file 3: Table S2. BLAST2GO PGSC pipeline output. Additional file 4: Table S3. OrthoMCL-UniProt PGSC pipeline output. Additional file 5: Table S4. Phytozome PGSC pipeline output. Additional file 6: Table S5. Tri_BLAST PGSC pipeline output. Additional file 7: Table S6. Tri_HMM PGSC pipeline output. Additional file 8: Table S7. BioMart ITAG pipeline output. Additional file 9: Table S8. BLAST2GO ITAG pipeline output. Additional file 10: Table S9. OrthoMCL-UniProt ITAG pipeline output. Additional file 11: Table S10. Phytozome ITAG pipeline output. Additional file 12: Table S11. Tri_BLAST ITAG pipeline output. Additional file 13: Table S12. Tri_HMM ITAG pipeline output. Additional file 14: Table S13. Potato gold standard genes with literature references. Additional file 15: Table S14. PGSC gold standard. Additional file 16: Table S15. ITAG gold standard. Additional file 17: Table S16. PGSC ensemble output with k = 2. Additional file 18: Table S17. ITAG ensemble output with k = 2. Additional file 19:
**GO annotation file based on ensemble k = 2 for JHI Solanum tuberosum 60 k v1 microarray (ArrayExpress ID: E-MTAB-1655).**

## Abbreviations

GO: Gene ontology
PGSC: Potato genome sequencing consortium
ITAG: International tomato annotation group
CC: Cellular component
MF: Molecular function
BP: Biological process
NGPG: Number of GO terms per gene
