# Supplementary material for: Evaluation and integration of functional annotation pipelines for newly sequenced organisms: the potato genome as a test case
Source: BMC Plant Biol. 2014 Dec 5;14:329. doi: 10.1186/s12870-014-0329-9 (PMC4274702; doi:10.1186/s12870-014-0329-9)

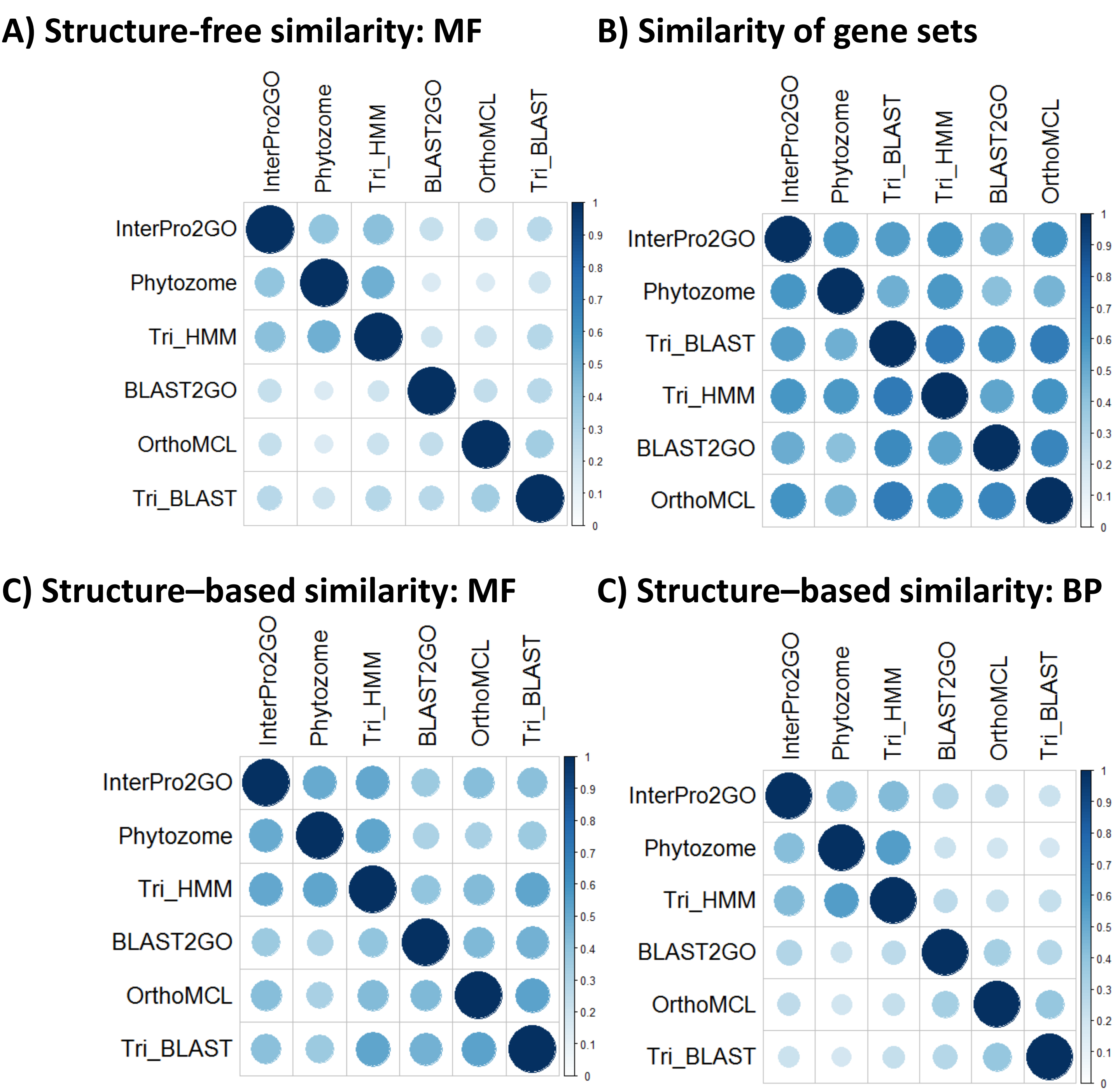

Figure S1. ITAG pipeline similarity. Similarity of annotation pipelines of the ITAG data. Each similarity matrix shows all pairwise similarities between the pipelines. (A) Structure-free similarity of the MF predictions of the pipelines. The similarity is calculated using the Jaccard coefficient. (B) Jaccard similarity of the gene sets covered by each pipeline. (C) GO-based similarity between the MF predictions of the pipelines. Unlike (A), the calculation here used the GODAG to quantify the similarity of the predictions (see Materials and Methods). (D) GO-based similarity between the BP predictions of the pipelines

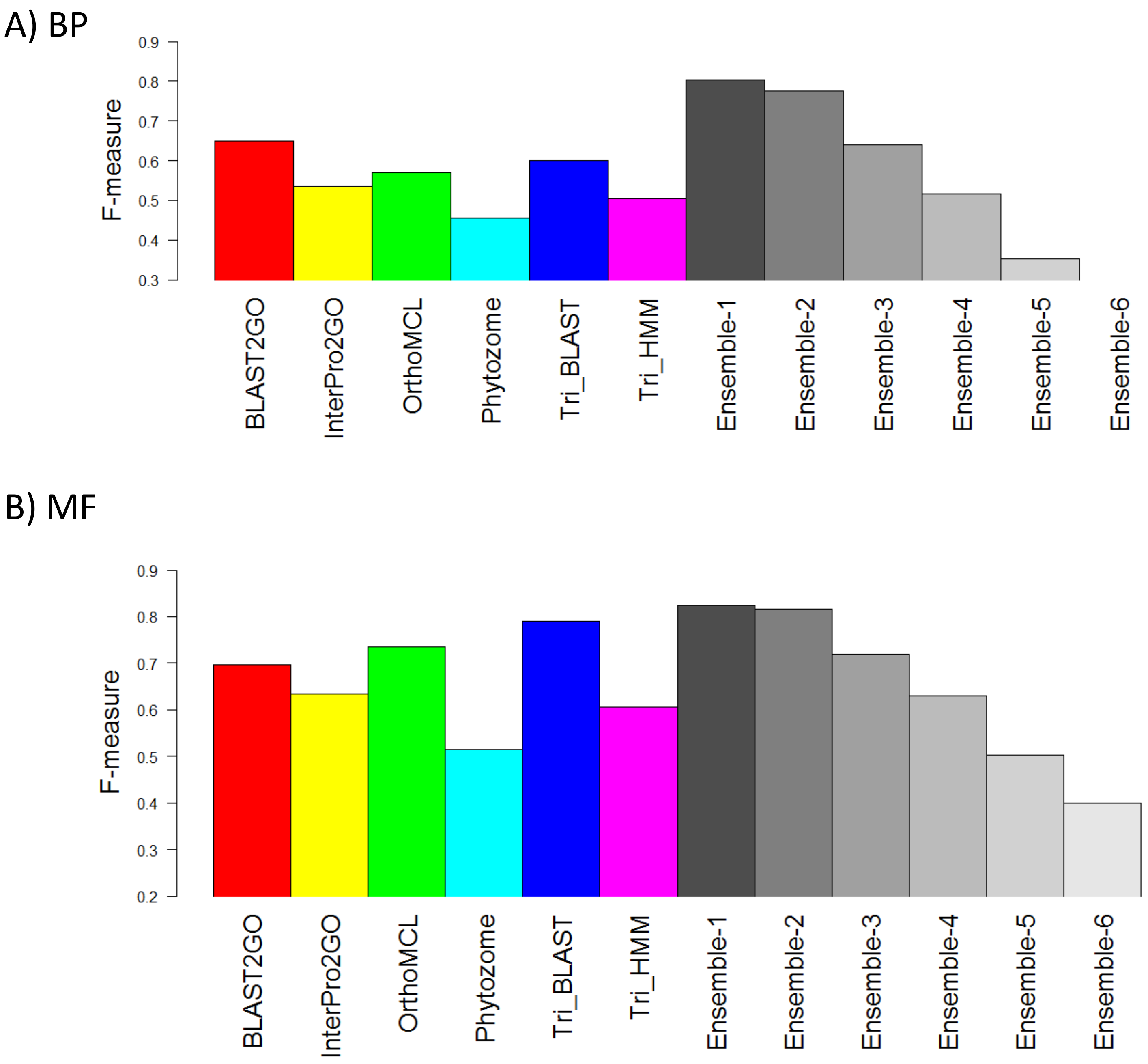

Figure S2. ITAG Gold Standard validation. ITAG Gold Standard validation. For each annotation method (i.e., a pipeline and a variant of the ensemble algorithm) the F-measure of the Gold Standard validation is shown, see Materials and Methods for a full description of the F-measure calculation. A score of 1 means a perfect agreement between an annotation flow and the Gold Standard. A score close to zero means that the annotation flow is not in line with the Gold Standard. (A) F-measure of the BP annotations. (B) F-measure of the MF annotations. The results show that both in BP and MF the ensemble algorithm improves the results considerably when k is 1 or 2.

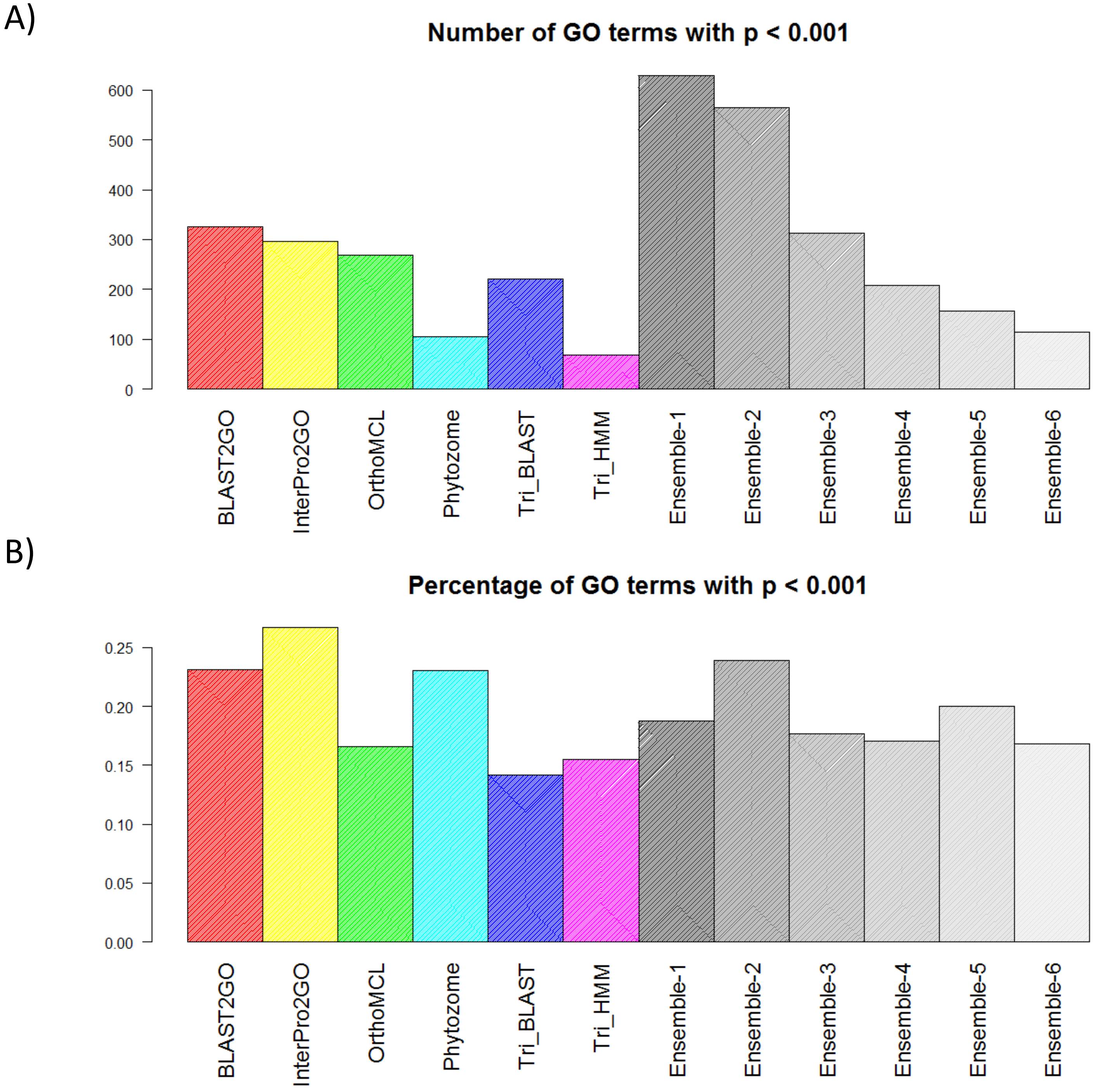

Figure S3. ITAG Gene Expression validation. ITAG coexpression validation. Given a set of genes linked to a biological process by a specific annotation method (i.e., the pipelines or a variant of the ensemble algorithm) the average co-expression of the genes was compared to that of random gene sets. For each annotation method the number of GO terms with p < 0:001 (A), and the percentage of GO terms with p < 0:001 (B) were calculated. The results show that the ensemble algorithm with k = 2 has a similar percentage of significant GO terms compared to the single pipelines. However, it provides > 1:6 fold more significant GO terms compared to the best single pipeline (BLAST2GO).

Supplemental Method 1. Composing the Gold Standard

The combined output of BLAST2GO, Trinotate_BLAST, Trinotate_HMM, Phytozome and OrthoMCL pipelines was checked against the set of identifiers of genes with known functional characterization. Only results with genes matching one in the set were processed. In order to manually asses them we added matching GO names and genes descriptions. Then we assigned scores to the results: 3 was assigned if the annotation was correct, 1 if it was not, 2 if we were not able to say (neutral). The results with score 3 compose the Gold Standard. Figure 1 presents the initial assessment of pipelines outputs.

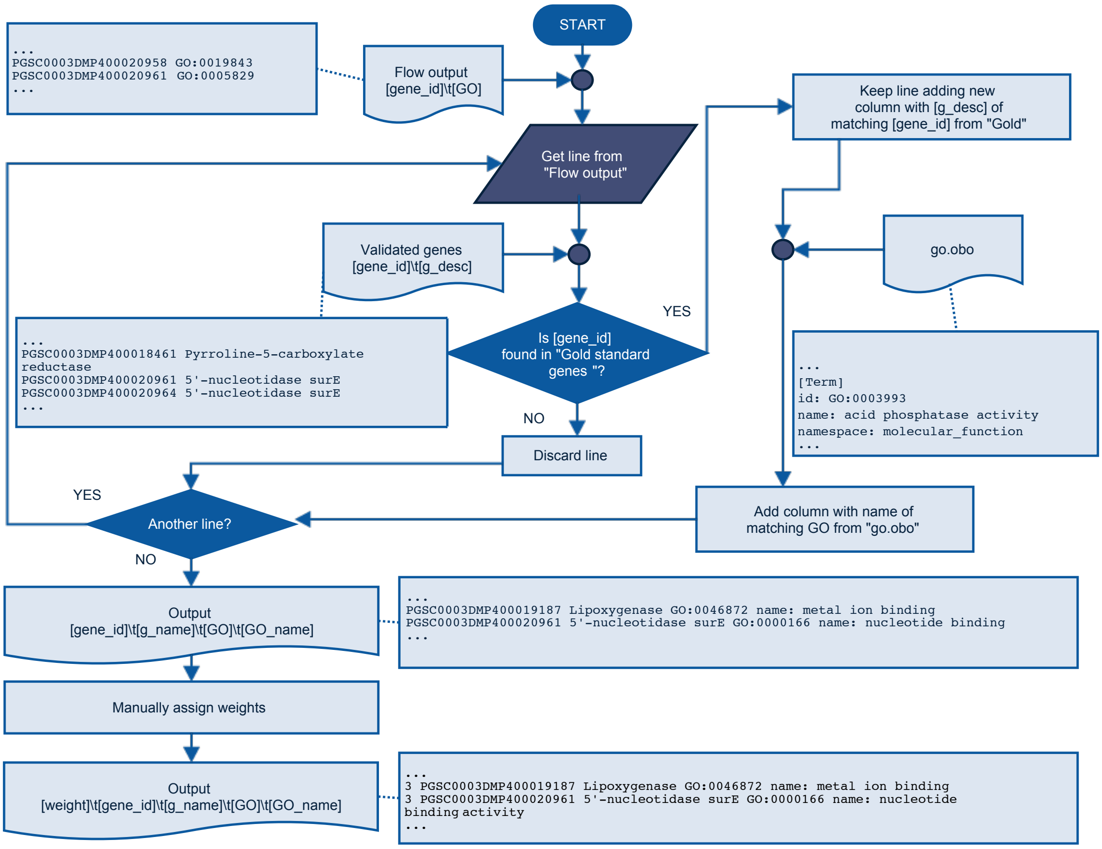

Fig. 1. Flowchart presenting the initial assessment of pipelines outputs.

Bash scripts `runGoldGenesMining_PGSC.sh` and `runGoldGenesMining_ITAG.sh` search parsed pipeline output for the results with genes matching any of genes with experimental validation in the literature. Names of GO terms and descriptions of genes are added to the results, to make the manual assessment easier.

1 SCRIPTS

`runGoldGenesMining_PGSC.sh`

```
#!/bin/bash
## Agnieszka Danek 2013
##
## Inputs:
## 1. [file_with_validated_gene_names] [gene_ID]\t[name]
## 2. [parsed_flow_output] formatted as [gene_ID]\t[GO]
## 3. [go.obo_file] go.obo.txt file should be available
##
### USAGE ###
## ./runGoldGenesMining_PGSC.sh [file_with_validated_gene_names] [parsed_flow_output]
    [go.obo_file]
```

```
## Required format of input files:
## 1. file_with_validated_gene_names:
## [gene_ID]\t[name]
## 2. parsed_flow_output:
## [gene_ID]\t[GO]
## 3. Standard "go.obo" file
##
## All files should be placed in the same (working) directory.
##
## The output file is placed in the "RESULTS" directory (created if doesn't exist).
## Its name is a concatenation of names first and second input files, with "-" between them and
    ".out" at the end:
## "RESULTS/[file_with_validated_gene_names]-[parsed_flow_output].out"

gold_file=$1
in_res=$2

#get file with SG ids only
awk '// {print $1} ' $gold_file > $gold_file.IDs

#search for SG in flow output
grep -f $gold_file.IDs $in_res > $in_res.gold

#remove file with SG ids
rm $gold_file.IDs

#add gene name/specification from go.obo.txt file according to go
while IFS=$'\t' read -r -a myArray
do
      id=`echo ${myArray[0]}`
      go=`echo ${myArray[1]}`
      awk -v i="$id" -v pat="$go" 'BEGIN{c=0} // {if(c==1){print i"\t"pat"\t"$0; c=0}} $1~/^id:/
          && $2~pat {c=1;} ' $3
done < $in_res.gold > $in_res.gold.oboNames

#add gene name/specification from "gold" according to gene id
while IFS=$'\t' read -r -a myArray
do
      id=`echo ${myArray[0]}`
      go=`echo ${myArray[1]}`
      name=`echo ${myArray[2]}`

      awk -v i="$id" -v g="$go" -v n="$name" 'BEGIN{c=0} $1~i {printf
          i"\t";{for(j=2;j<=NF;++j)printf $j" "} print"\t"g"\t"n} ' $gold_file

done < $in_res.gold.oboNames > $in_res.gold.oboNames.goldNames

rm $in_res.gold.oboNames
rm $in_res.gold

#make directory RESULTS (if does not exist)
if [ ! -d RESULTS ]
then
 mkdir RESULTS
fi

#Move output results to RESULTS directory.
#Name is a concatenation of names of inputs
    ("[name_of_file_with_gold_gene_names]-[name_of_file_with_flow_results].out")
cp $in_res.gold.oboNames.goldNames RESULTS/$gold_file-$in_res.out
rm $in_res.gold.oboNames.goldNames
```

---

runGoldGenesMining_PGSC.sh

```
#!/bin/bash
## Agnieszka Danek 2013
##
## inputs:
```

```
## 1.[name_of_file_with_gold_gene_names] [gene_ID]\t[PGSC_id]\t[name]
## 2.[name_of_file_with_flow_results] formatted as [gene_ID]\t[GO]
## Additionally go.obo.txt file should be available in the running directory
##
### USAGE ###
## ./runGoldGenesMining_ITAG.sh [file_with_validated_gene_names] [parsed_flow_output]
     [go.obo_file]
##
## Required format of input files:
## 1. file_with_validated_gene_names:
## [gene_ID]\t[name]
## 2. parsed_flow_output:
## [gene_ID]\t[GO]
## 3. Standard "go.obo" file
##
## All files should be placed in the same (working) directory.
##
## The output file is placed in the "RESULTS" directory (created if doesn't exist).
## Its name is a concatenation of names first and second input files, with "-" between them and
     ".out" at the end:
## "RESULTS/[file_with_validated_gene_names]-[parsed_flow_output].out"

gold_file=$1
in_res=$2

#get file with SG ids only
awk '// {print $1} ' $gold_file > $gold_file.IDs

#search for SG in flow output
grep -f $gold_file.IDs $in_res > $in_res.gold

#remove file with SG ids
rm $gold_file.IDs

#add gene name/specification from go.obo.txt file according to gene id and go
while IFS=$'\t' read -r -a myArray
do
        id=`echo ${myArray[0]}`
        go=`echo ${myArray[1]}`
        awk -v i="$id" -v pat="$go" 'BEGIN{c=0} // {if(c==1){print i"\t"pat"\t"$0; c=0}} $1~/^id:/
             && $2~pat {c=1;} ' go.obo.txt
done < $in_res.gold > $in_res.gold.oboNames

#add gene name/specification from "gold" according to gene id
while IFS=$'\t' read -r -a myArray
do
        id=`echo ${myArray[0]}`
        go=`echo ${myArray[1]}`
        name=`echo ${myArray[2]}`

        awk -v i="$id" -v g="$go" -v n="$name" 'BEGIN{c=0} $1~i {printf i"\t";printf
             $2"\t";{for(j=3;j<=NF;++j)printf $j" "} print"\t"g"\t"n} ' $gold_file

done < $in_res.gold.oboNames > $in_res.gold.oboNames.goldNames

rm $in_res.gold.oboNames
rm $in_res.gold

if [ ! -d RESULTS ]
then
 mkdir RESULTS
fi

#Move output results to RESULTS directory.
#Name is a concatenation of names of inputs
     ("[name_of_file_with_gold_gene_names]-[name_of_file_with_flow_results].out")
cp $in_res.gold.oboNames.goldNames RESULTS/$gold_file-$in_res.out
rm $in_res.gold.oboNames.goldNames
```

Supplemental Method 2. The OrthoMCL pipeline

1 RUN BLAST FOR 18 COMPLETE PLANT PROTEOMES AGAINST UNIPROT

As OrthoMCL does not assign GO terms to genes, we did a BLASTP search of our protein sequences against the UniProt database, and get the BLASTP output in a tabular format.

Here is an example of the first lines of the output file for the A. thaliana proteome (we have one BLAST output per proteome):

```
atha|AT4G32375.1|PACid:19647937 sp|Q9LW07|PGLR3_ARATH 38.68 393 213 10 5 382 42 421 1e-83 272
atha|AT4G32375.1|PACid:19647937 sp|P05117|PGLR_SOLLC 38.73 346 195 7 4 342 93 428 4e-70 236
atha|AT4G32375.1|PACid:19647937 sp|P35336|PGLR_ACTDE 36.86 331 193 6 5 328 107 428 6e-68 231
atha|AT4G32375.1|PACid:19647937 sp|Q02096|PGLR_PERAE 37.39 337 195 5 5 334 102 429 6e-68 231
atha|AT4G32375.1|PACid:19647937 sp|P43212|PGLR2_CRYJA 37.06 340 194 6 5 334 77 406 2e-67 231
```

All the BLAST output files must be placed in the same directory, and should have the extension .blast (for example "Athaliana_Uniprot.blast").

2 RETRIEVE GO TERMS FOR ALL ANNOTATED UNIPROT ENTRIES

We then retrieve GO terms from the UniProt entries of the 10 best UniProt hits and create another tabular file using the java program AllBio_GetGOTermsFromSwissProt. Program call:

```
javac AllBio_GetGOTermsFromSwissProt.java (program compilation)
java AllBio_GetGOTermsFromSwissProt -i /path_to_the_Swissprot_db/  -o /path_to_the_out_file/
```

The first column is the UniProt ID of the hit; the second column contains a list of GO terms and GO IDs associated with this hit, separated by ; (semicolon). In this example we call it Swissprot_GO.txt. The first lines of this file look like this:

```
1A1D_PYRAB GO:0008660; F:1-aminocyclopropane-1-carboxylate deaminase activity;
      IEA:UniProtKB-EC.GO:0008152; P:metabolic process; IEA:GOC.
1A1D_PYRFU GO:0008660; F:1-aminocyclopropane-1-carboxylate deaminase activity;
      IEA:UniProtKB-EC.GO:0008152; P:metabolic process; IEA:GOC.
1A1D_PYRHO GO:0008660; F:1-aminocyclopropane-1-carboxylate deaminase activity;
      IEA:UniProtKB-EC.GO:0008152; P:metabolic process; IEA:GOC.
3MGH_AERPE GO:0003905; F:alkylbase DNA N-glycosylase activity; IEA:InterPro.GO:0003677;
      F:DNA binding; IEA:InterPro.GO:0006284; P:base-excision repair; IEA:InterPro.
```

3 ANNOTATE 18 COMPLETE PROTEOMES BY GO TERMS FROM THEIR TEN BEST BLAST HITS

We now need to assign the UniProt GO terms to our PGSC and ITAG proteins in the OrthoMCL clusters. This is a 2-step process. First we assign GO terms to all the protein sequences from the 18 proteomes. We run the Perl script assign_GO_to_18proteomes.pl. You need to run it in the directory where your BLAST output files are. The arguments to run it are the file Swissprot_GO.txt and the output file of this script (we will call it 18proteomes_GO.txt). Run the script in the terminal as followed:

```
$ perl assign_GO_to_18proteomes.pl Swissprot_GO.txt  18proteomes_GO.txt
```

The first lines of 18proteomes_GO.txt look like this:

```
acoe|Aquca_001_00003.1#PACid:22042945 GO:0008270
acoe|Aquca_001_00004.1#PACid:22042917 GO:0003676 GO:0008270
acoe|Aquca_001_00005.1#PACid:22043803 GO:0005524 GO:0004674
acoe|Aquca_001_00007.1#PACid:22043907 GO:0016787 GO:0008531 GO:0009231
```

4 SCRIPTS

assign_GO_to_18proteomes.pl

```
#!/usr/bin/perl -w
use strict;

my $all = $ARGV[0]; #the GO <> Uniprot tabular file
my $out = $ARGV[1]; #the name of the output file

my ($x,@sp,@spname,$id,%go);
open(ALL, "< $all");
while(<ALL>){
   chomp;
   @spname = split(/\t/,$_);
   if ($#spname>0){
```

```
        $id = $spname[0];
        @sp = split(/\./,$spname[1]);
        foreach $x (@sp){
            if ($x =~ /(GO:\d+)/){
                $go{$id} .= "\t$1";
            }
        }
    }
}
close(ALL);

my @file = glob('*.blast'); #here all our blast output have the extension .blast

my ($y,$n,%map);
$id=();
foreach $x (@file){
    open (IN, "< $x");
    while(<IN>){
        chomp;
        @sp = split(/\t/,$_);
        if ((!(defined($id))) || ($id ne $sp[0])){
            $id = $sp[0];
            $n = 1;$y = 0;
        }
        $sp[1] =~ /^\w+\|\w+\|(\w+_\w+)$/;
        if (defined($go{$1})){
            if (($y == 0) && ($n <= 10)){
                $map{$id} = $go{$1};
                $y = 1; $n=1;
            }
        }
        else {
            $n++;
        }
    }
}
open(OUT, "> $out");
foreach my $k (sort keys(%map)){
    print OUT "$k\t$map{$k}\n";
}
exit(0);
```

AllBio_GetGOtermsForClusterMembers.java

```
import java.io.BufferedReader;
import java.io.File;
import java.io.FileReader;
import java.io.IOException;
import java.io.PrintWriter;
import java.util.HashSet;
import java.util.Hashtable;

/**
 * This is a program that takes
 * the input files are:
 * 1. the output file of orthomcl
 * 2. tab delimited file of a protein_name and its GO terms
 *
 * the output is:
 * a file with one protein_name and one GO term inferred from the orthomcl cluster
 * @author Tatyana
 *
 */
public class AllBio_GetGOtermsForClusterMembers {

      private Hashtable<String, HashSet<String>> proteinName_GOIds; //content of the protein
          name to GO terms mapping
      private Hashtable<String, HashSet<String>> itag_GOIds;
```

```java
private Hashtable<String, HashSet<String>> pgsc_GOIds;

/**
 * Constructor to control the program flow
 * @param orthomclOut
 *          input - orthoMCL results file
 * @param GO_mappingFile
 *          input - tab delimeted mapping file of a protein id and its assiciated GO terms
 * @param itag
 *          output - ITAG proteins and their GO annotations
 * @param pgsc
 *          output - PGSC proteins and their GO annotations
 * @throws IOException
 */
public AllBio_GetGOtermsForClusterMembers(String orthomclOut, String GO_mappingFile,
    String itag, String pgsc) throws IOException{

      //get GO terms for every protein in the 18 organisms from the GO mapping file
      proteinName_GOIds=new Hashtable<String, HashSet<String>>();
      getGOtermsMappingFile(GO_mappingFile);
      System.out.println("number of proteins read from "+GO_mappingFile+" is
          "+proteinName_GOIds.size());

      //now go through the clusters of OrthoMCL
      itag_GOIds=new Hashtable<String, HashSet<String>>();
      pgsc_GOIds=new Hashtable<String, HashSet<String>>();
      readOrthoMCLFile(orthomclOut);

      //print the two result files; one for itag and one for pgsc
      printResults(itag, pgsc);
}

/**
 * Print the result files
 * @param itagOutFile
 *                output file for itag proteins
 * @param pgscOutFile
 *                output file for pgsc proteins
 * @throws IOException
 */
public void printResults(String itagOutFile, String pgscOutFile) throws IOException{
      PrintWriter pw_itag = new PrintWriter(new File(itagOutFile));
      PrintWriter pw_pgsc = new PrintWriter(new File(pgscOutFile));
      for(String itagId: itag_GOIds.keySet()){
            for(String go : itag_GOIds.get(itagId)){
                  pw_itag.println(itagId+"\t"+go);
                  //          System.out.println(itagId+"\t"+go);
            }
      }
      for(String pgsc : pgsc_GOIds.keySet()){
            for(String go: pgsc_GOIds.get(pgsc)){
                  pw_pgsc.println(pgsc+"\t"+go);
                  //          System.out.println(pgsc+"\t"+go);
            }
      }
      pw_itag.close();
      pw_pgsc.close();
}

/**
 * Go through the clusters of OrthoMCL result file
 * @param orthomclOut
 *                orthomcl results file
 * @throws IOException
 */
public void readOrthoMCLFile(String orthomclOut) throws IOException{
      BufferedReader br = new BufferedReader(new FileReader(orthomclOut));
      String line="";
      String [] split_line;
      HashSet<String> GOterms;
```

```
		HashSet<String> itag_prots;
		HashSet<String> pgsc_prots;
		HashSet<String> GOlistFromMappingFile;
		HashSet<String> helpGOHash;
		HashSet<String> prevGOTerms=null;
		while((line=br.readLine())!=null){
			line=line.substring(line.indexOf(": "));
			split_line=line.split(" ");
			GOterms=new HashSet<String>();
			itag_prots=new HashSet<String>();
			pgsc_prots=new HashSet<String>();
			GOlistFromMappingFile=new HashSet<String>();
			for(int i=0; i<split_line.length;i++){
				if(proteinName_GOIds.containsKey(split_line[i])){
					GOlistFromMappingFile=proteinName_GOIds.get(split_line[i]);
					for(String id:GOlistFromMappingFile){
						GOterms.add(id);
					}
				}
				if(split_line[i].startsWith("stub")){
					itag_prots.add(split_line[i]);
				} else if(split_line[i].startsWith("pgsc")){
					pgsc_prots.add(split_line[i]);
				}
			}
			for(String itagId: itag_prots){
				if(itag_GOIds.containsKey(itagId)){
					helpGOHash=itag_GOIds.get(itagId);
					for(String go : GOterms){
						helpGOHash.add(go);
					}
				} else {
					helpGOHash=GOterms;
				}
				if(itag_GOIds.containsKey(itagId)){
					prevGOTerms=itag_GOIds.get(itagId);
					for(String go: prevGOTerms){
						GOterms.add(go);
					}
				}
				itag_GOIds.put(itagId, GOterms);
			}

			for(String pgscId: pgsc_prots){
				if(pgsc_GOIds.containsKey(pgscId)){
					helpGOHash=pgsc_GOIds.get(pgscId);
					for(String go: GOterms){
						helpGOHash.add(go);
					}
				} else {
					helpGOHash=GOterms;
				}
				if(pgsc_GOIds.containsKey(pgscId)){
					prevGOTerms=pgsc_GOIds.get(pgscId);
					for(String go: prevGOTerms){
						GOterms.add(go);
					}
				}
				pgsc_GOIds.put(pgscId, GOterms);
			}
		}
		br.close();
	}

	/**
	 * Go through the GO mapping file and keep the mapping in memory
	 * @param GO_mappingFile
	 *			tab delimited text file with >=1 GO term per protein
	 * @throws IOException
	 */
```

```java
public void getGOtermsMappingFile(String GO_mappingFile) throws IOException{
	BufferedReader br = new BufferedReader(new FileReader(GO_mappingFile));
	String line="";
	String [] splitLine;
	HashSet<String> GOlist;
	while((line=br.readLine())!=null){
		splitLine=line.split("\t");
		GOlist=new HashSet<String>();
		for(int i=1; i<splitLine.length;i++){
			GOlist.add(splitLine[i]);
		}
		proteinName_GOIds.put(splitLine[0], GOlist); //one protein can have >=1 GO term
	}
	br.close();
}

public static void printHelp(){
	System.err.println("DESCRIPTION");
	System.err.println("Program to assign GO terms to ITAG and PGSC proteins based on
	    their OrthoMCL clusters.\n");
	System.err.println("COMPILATION");
	System.err.println("javac AllBio_GetGOtermsForClusterMembers.java\n");
	System.err.println("USAGE");
	System.err.println("java AllBio_GetGOTermsFromSwissProt -o <input_file> -g
	    <input_file> -i <output_file> -p <output_file>\n");
	System.err.println("MANDATORY");
	System.err.println("-o\t<file>");
	System.err.println("\t\tOrthoMCL output file containing clusters for ITAG and PGSC
	    proteins.");
	System.err.println("-g\t<file>");
	System.err.println("\t\tTab separated file with GO annotations for every protein in
	    18 complete plant proteomes.\n");
	System.err.println("-i\t<file>");
	System.err.println("\t\tOutput file for ITAG proteins containing GO assignments
	    based on OrthoMCL clusters\n");
	System.err.println("-p\t<file>");
	System.err.println("\t\tOutput file for PGSC proteins containing GO assignments
	    based on OrthoMCL clusters\n");
	System.err.println("OPTIONS");
	System.err.println("-h");
	System.err.println("\t\tPrints this help.\n");
	System.err.println("EXAMPLE FILES");
	System.err.println("OrthoMCL result may have following format:");
	System.err.println("clus1: prot1 stub1 pgsc1 prot2 prot3");
	System.err.println("clus2: prot1 prot2 pgsc1");
	System.err.println("clus3: pgsc2");
	System.err.println("GO mapping file may have following format:");
	System.err.println("prot1\tGO1 GO2");
	System.err.println("prot2");
	System.err.println("pgsc1\tGO3 GO1");
	System.err.println("pgsc2\tGO4");
	System.err.println("stub1\tGO5");
	System.err.println("The result file for ITAG will then look like:");
	System.err.println("stub1\tGO5");
	System.err.println("stub1\tGO1");
	System.err.println("stub1\tGO2");
	System.err.println("stub1\tGO3");
	System.err.println("The result file for PGSC will then look like:");
	System.err.println("pgsc2\tGO4");
	System.err.println("pgsc1\tGO1");
	System.err.println("pgsc1\tGO2");
	System.err.println("pgsc1\tGO3");
	System.err.println("pgsc1\tGO5");
}

public static void main(String[] args) throws IOException{
			String orthomclOut="";
			boolean isOrthoMCLDefined=false;
			String GO_mappingFile="";
			boolean isGO_mappingFileDefined=false;
```

```
			String itag="";
			boolean isITAGDefined=false;
			String pgsc="";
			boolean isPGSCDefined=false;
			for (int i = 0; i < args.length; i = i + 2) {
				if (args[i].equals("-o")) {
					orthomclOut = args[i + 1];
					isOrthoMCLDefined=true;
				}
				if (args[i].equals("-g")) {
					GO_mappingFile = args[i + 1];
					isGO_mappingFileDefined=true;
				}
				if (args[i].equals("-i")) {
					itag = args[i + 1];
					isITAGDefined=true;
				}
				if (args[i].equals("-p")) {
					pgsc = args[i + 1];
					isPGSCDefined=true;
				}
				if(args[i].equals("-h")){
					printHelp();
					System.exit(0);
				}
			}

			//throw error messages if input/output files are missing
					if(isOrthoMCLDefined==false){
						System.err.println("Input OrthoMCL file is
						    missing.\n");
						printHelp();
						System.exit(1);
					}
					if(isGO_mappingFileDefined==false){
						System.err.println("Input GO mapping file is
						    missing.\n");
						printHelp();
						System.exit(1);
					}
					if(isITAGDefined==false){
						System.err.println("Output ITAG file is missing.\n");
						printHelp();
						System.exit(1);
					}
					if(isPGSCDefined==false){
						System.err.println("Output PGSC file is missing.\n");
						printHelp();
						System.exit(1);
					}
		AllBio_GetGOtermsForClusterMembers getGO = new
		    AllBio_GetGOtermsForClusterMembers(orthomclOut, GO_mappingFile, itag, pgsc);
	}
}
```

AllBio_GetGOTermsFromSwissProt.java

```
/**
 * This is a class to extract GO terms for all proteins in Swiss-Prot
 * Input: Swiss-Prot database in the dat format.
 * Output: tab delimeted file with protein id and its associated GO terms:
     protein_id\tGO_term1;GO_term2
 *
 * @author Tatyana
 *
 */
import java.io.BufferedReader;
import java.io.FileReader;
```

```java
import java.io.FileWriter;
import java.io.IOException;
import java.io.PrintWriter;
import java.util.regex.Matcher;
import java.util.regex.Pattern;

public class AllBio_GetGOTermsFromSwissProt {
      private final String ID = "ID ";
      private final String GO="DR GO; ";
      private final String END = "//";
      private int noIds_all = 0;
      private int noIds_withGOTerms = 0;

      /**
       * Constructor to read the dat file(s) and get GO terms
       *
       * @param datFile
       *         a plain swissprot dat file
       * @param spId_out
       *         file to save the ids and their GO terms in a tab separated format
       */
      public AllBio_GetGOTermsFromSwissProt(String[] datFiles, String outFile) throws
          IOException {
            PrintWriter pw = new PrintWriter(new FileWriter(outFile));
            for (int i = 0; i < datFiles.length; i++) {
                  System.out.println("reading "+datFiles[i]);
                  readDatFile(datFiles[i], pw);
            }
            pw.close();
            System.out.println("number ids visited: "+noIds_all);
            System.out.println("number ids with GO terms: "+noIds_withGOTerms);
      }

      /**
       * Method to read a dat file. If there is a GO term associated with an entry found
       * then it is being printed via PrintWriter stream
       * @param datFile
       *                 input file
       * @param pw
       *                 writes the result data
       * @throws IOException
       */
      public void readDatFile(String datFile, PrintWriter pw) throws IOException {
            BufferedReader br = new BufferedReader(new FileReader(datFile));
            String line = "";
            String id = "";
            String go = "";
            boolean isFoundAC=false;

            while ((line = br.readLine()) != null) {
                  //parse the id
                  if(line.startsWith(ID)){
                        Pattern pat =
                            Pattern.compile("^ID\\s+([a-zA-Z0-9]+_[a-zA-Z0-9]+)\\s+.+$");
                        Matcher mat = pat.matcher(line);
                        if (mat.find()) {
                              id = mat.group(1);
                              isFoundAC=true;
                              noIds_all++;
                        }
                  }
                  //parse GO terms
                  else if(line.startsWith(GO) && isFoundAC==true){
                        go = go.concat(line.replace(GO, ""));

                  }
                  //if reached the end of an entry, then write the result if it is available
                  else if (line.startsWith(END) && isFoundAC==true){
                        if(!go.equals("")){
                              //    System.out.println(">"+id+"\t"+go);
```

```
                        pw.println(id+"\t"+go);
                        noIds_withGOTerms++;
                    }
                    isFoundAC=false;
                    go="";
                }
            }
            br.close();
        }

        public static void printHelp(){
            System.err.println("DESCRIPTION");
            System.err.println("Program to print for every protein entry in Swiss-Prot its
                associated GO term(s).\n");
            System.err.println("COMPILATION");
            System.err.println("javac AllBio_GetGOTermsFromSwissProt.java\n");
            System.err.println("USAGE");
            System.err.println("java AllBio_GetGOTermsFromSwissProt -i <input_file> -o
                <output_file>\n");
            System.err.println("MANDATORY");
            System.err.println("-i\t<file>");
            System.err.println("\t\tSwiss-Prot file in dat format. User can provide more than
                one dat file by separating them with a '#' (hash).");
            System.err.println("-o\t<file>");
            System.err.println("\t\tOutput file.\n");
            System.err.println("OPTIONS");
            System.err.println("-h");
            System.err.println("\t\tPrints this help.\n");
        }

        public static void main(String[] args) throws IOException {
            String outFile="";
            String[] datFiles = null;

            boolean isInputDefined=false;
            boolean isOutputDefined=false;

            //parse the input
            for (int i = 0; i < args.length; i = i + 2) {
                if (args[i].equals("-i")) {
                    datFiles = args[i + 1].split("#");
                    isInputDefined=true;
                }
                if (args[i].equals("-o")) {
                    outFile = args[i + 1];
                    isOutputDefined=true;
                }
                if(args[i].equals("-h")){
                    printHelp();
                    System.exit(0);
                }
            }

            //throw error messages if input/output files are missing
            if(isInputDefined==false){
                System.err.println("Input file(s) is(are) missing.\n");
                printHelp();
                System.exit(1);
            }
            if(isOutputDefined==false){
                System.err.println("Output file is missing.\n");
                printHelp();
                System.exit(1);
            }
            AllBio_GetGOTermsFromSwissProt list = new AllBio_GetGOTermsFromSwissProt(datFiles,
                outFile);
        }
}
```

Supplemental Method 3. The Trinotate pipeline

Here we describe the necessary steps to perform the annotation with Trinotate. We used default settings everywhere to annotate the input data with NCBI-BLAST (with SwissProt database) and HMMER tool (with Pfam database). Trinotate uses specific releases of these databases. The results were collected with Trinotate, according to the guidelines (`http://trinotate.sourceforge.net`). We performed the annotation for two sets of gene models:

- ITAG
  File with coding sequences was used as a main input:
  `potato.Sotub.cds.itag.v1.fasta`
  (`https://bioinformatics.psb.ugent.be/gdb/potato/potato.Sotub.cds.itag.v1.fasta.bz2`)

- PGSC
  File with peptide representatives was used as a main input:
  `PGSC_DM_v3.4_pep_representative.fasta`
  (`http://potato.plantbiology.msu.edu/data/PGSC_DM_v3.4_pep_representative.fasta.zip`)
  File with coding sequences was used only to populate the sqlite database with results:
  `PGSC_DM_v3.4_cds_representative.fasta`
  (`http://potato.plantbiology.msu.edu/data/PGSC_DM_v3.4_cds_representative.fasta.zip`)

For that reason some comments are specific to these inputs. In general, input with coding sequences is reffered to as transcripts.fasta, while input with peptide representatives is reffered to as peptide.fasta. As trinotate is designed to annotate transcriptts produced by Trinity, some additional pre-processing is necessary, if the input comes from other source.

All scripts can be run in UNIX environment. Comments includes additional hints on running the pipeline.

1 DOWNLOADING REQUIRED PROGRAMS AND DATABASES

```
# Download trinotate (version 20130706 was used)
wget http://downloads.sourceforge.net/project/trinotate/trinotate_r20130706.tgz
# Download trinity to use some of its tools (transdecoder)
wget http://sourceforge.net/projects/trinityrnaseq/files/trinityrnaseq_r2013-02-25.tgz
# Download ncbi-blast
wget ftp://ftp.ncbi.nlm.nih.gov/blast/executables/LATEST//ncbi-blast-2.2.28+-x64-linux.tar.gz
# Download swissprot database
wget http://sourceforge.net/projects/trinotate/files/TRINOTATE_RESOURCES/uniprot_sprot.fasta.gz
# Download hmmer tool
wget ftp://selab.janelia.org/pub/software/hmmer3/3.1b1/hmmer-3.1b1-linux-intel-x86_64.tar.gz
# Download pfam-A database
wget http://sourceforge.net/projects/trinotate/files/TRINOTATE_RESOURCES/Pfam-A.hmm.gz
# Download Trinotate regenerated sqlite database.
wget http://sourceforge.net/projects/trinotate/files/TRINOTATE_RESOURCES/
/TrinotateResources-20130704/TrinotateSqlite.sprot.20130704.db.gz
# We used 20130704 version of sqlite database.
# If not available, the newest version can be used (see below)
# In that case all future steps with sqlite database should be alerted accordingly
wget http://sourceforge.net/projects/trinotate/files/Trinotate_r20131110.tar.gz
```

2 DECOMPRESSING TOOLS AND DATABASES

```
# Decompress trinotate
tar -xf trinotate_r20130706.tgz
# Decompress trinity
tar -zxf trinityrnaseq_r2013-02-25.tgz
# Decompress ncbi-blast
tar -zxvpf ncbi-blast-2.2.28+-x64-linux.tar.gz
# Decompress swissprot
gunzip uniprot_sprot.fasta.gz
# Decompress hmmer
tar -zxf hmmer-3.1b1-linux-intel-x86_64.tar.gz
# Decompress Pfam-A
gunzip pfam-A.hmm.gz
# Deocmpress sqlite trinotate database
gunzip TrinotateSqlite.sprot.20130704.db.gz
```

3 BUILDING AND NECESSARY PREPROCESSING

```
# Build trinity - some errors may occur, but it's necessary that transcoder is made
cd trinityrnaseq_r2013-02-25
make
cd ..
# Configure and build Hmmer
cd hmmer-3.1b1-linux-intel-x86_64/
./configure
make
make check
cd ..
# Make blast database from swissprot.db
ncbi-blast-2.2.28+/bin/makeblastdb -in uniprot_sprot.fasta -dbtype prot
# Prepare Pfam-A.hmm for use with hmmscan
./hmmer-3.1b1-linux-intel-x86_64/binaries/hmmpress Pfam-A.hmm
```

4 PROCESSING

The input can be either coding sequences (transcripts.fasta) or peptide representatives (peptide.fasta). In case the input is transcripts.fasta, first step is necessary. With peptide.fasta available skip the first step.

```
### 1 ### Generate most likely Longest-ORF peptide candidates
./trinityrnaseq_r2013-02-25/trinity-plugins/transdecoder/transcripts_to_best_scoring_ORFs.pl
 -t transcripts.fasta
cp best_candidates.eclipsed_orfs_removed.pep peptide.fasta
### 2 ### Capturing BLAST Homologies
## adjust number of used threads to your machine (" -num_threads [NUMBER]")
ncbi-blast-2.2.28+/bin/blastp -query peptide.fasta -db uniprot_sprot.fasta -num_threads 16
 -max_target_seqs 1 -outfmt 6 > TrinotateBlast.out
### 3 ### Running HMMER to identify protein domains
./hmmer-3.1b1-linux-intel-x86_64/binaries/hmmscan --cpu 8 --domtblout TrinotatePFAM.out
 Pfam-A.hmm peptide.fasta > pfam.log
### 4 ### Trinotate: Loading Above Results into a Trinotate SQLite Database
## pre-step 4a: change TrinotatePFAM.out (loading didn't work without the change):
## remove last lines with comments (#)
## pre-step 4b' (specifically for ITAG data): create tab-delimeted file with
## "gene_id(tab)transcript_id" ("transcript_id(tab)transcript_id")
awk '/>/ {print $1"\t"$1}' transcripts.fasta | sed 's/>//g' > potato.gene_trans_map
## pre-step 4b'' (specifically for PGSC data): create tab-delimeted file with
## "gene_id(tab)transcript_id"
## As we used peptide representatives for PGSC and Trinotate needs
## transcripts.fasta to populate the database, we used transcripts.fasta for PGSC
## and matched the IDs, to create a required tab-delimeted file
sed 's/DMC/DMP/g' PGSC_DM_v3.4_cds_representative.fasta > transcripts.fasta
awk '/>/ {print $1"\t"$1}' transcripts.fasta | sed 's/>//g' > potato.gene_trans_map
## 4.1 ### create new database "Trinotate.sqlite"
cp TrinotateSqlite.sprot.20130704.db Trinotate.sqlite
## Populating the sqlite database
./trinotate_r20130706/Trinotate.pl init --gene_trans_map potato.gene_trans_map
  --transcript_fasta transcripts.fasta --transdecoder_pep peptide.fasta
## Load blast homologies
./trinotate_r20130706/Trinotate.pl LOAD_blast TrinotateBlast.out
## Load Pfam domain entries
./trinotate_r20130706/Trinotate.pl LOAD_pfam TrinotatePFAM.out
### 5 ### Trinotate: Output an Annotation Report
./trinotate_r20130706/Trinotate.pl report > trinotate_output.txt
```

5 EXTRACTION OF GO-GENE PAIRS

We used Python script that has a main script (`parse_trinotate_output.py`) and a method sub-script (`helper_methods.py`) to parse the `trinotate_output.txt` into two files, one with annotations made with NCBI-BLAST, one with annotations made with HMMER. Both scripts are available in Supplementary Materials. Format of each file:

```
[gene ID]\t[GO term]
```

6 SCRIPTS

parse_trinotate_output.py

```
# AUTHOR: David Amar
# August 2013

import re
import helper_methods
pfam2go = helper_methods.readMappingFile(path="pfam2go.txt",key_sep = "Pfam:|\s+",key_sep_ind=1)
trino_file = open("trinotate_output.txt")
trino_file.readline() # skip the header

out1 = open("trinotate_pfam2go_preds.txt","w")
out2 = open("trinotate_directGO_preds.txt","w")

# go over the output of trinotate, line by line
for l in trino_file:
   l = l.rstrip()
   arr = l.split("\t")
   gene = arr[0]

   ###### Analyze the pfam based prefictions #####
   # step 1. Get the association E-scores
   pfam_raw_info = arr[5]
   # The actual E-score always starts with "E:"
   score_regex = "E:"
   scores_raw = re.split(score_regex,pfam_raw_info)
   scores = []
   # The E-score of the association ends with ` (or when the line ends)
   for i in range(1,len(scores_raw)):
      scores.append(re.split(r'\^|`',scores_raw[i])[0])

   # step 2. Get the pfam ids for each association and get its score
   # Note: if a certain pfam id has more than one E-score, we keep the lowest
   pfam_col = re.split(r'\^|W|`',pfam_raw_info)
   # this hash keeps a mapping from pfam ids to their scores
   pfam = {}
   # this arguments keeps where we are in the scores list
   hit_counter = 0
   # a pfam id starts with "PF" and followed by numbers
   for p in pfam_col:
      if not re.search("^PF\d+",p):continue
      # trinotate's annotations has the pfam id then a dot then a number
      # to map pfam ids to GO terms we only need the prefix
      p = re.split(r'\.',p)[0]
      # get the minimal score
      pfam[p]= min(pfam.get(p,1),float(scores[hit_counter]))
      hit_counter +=1
   # this is a test for QA: if the pointer "hit_counter" did not
   # reach the last value in scores than we have an error
   # note that if the length of scores is lower than the number
   # of observed pfam ids then an exception will be thrown (i.e., the
   # run stops with an error)
   if hit_counter != len(scores): break

   # step 3.go over the pfam ids, map them to GO terms
   # and write the results to the output file (inclusing the scores)
   for p in pfam.keys():
      if not pfam2go.has_key(p):continue
      escore = pfam[p]
      for g in pfam2go.get(p):
         out1.write(gene+"\t"+g+"\t"+str(escore)+"\n")

   ###### Analyze the GO prefictions #####
   # here we analyze the column that directly predicts
   # GO terms for each gene

   # step 1. parse the go predictions
   curr_gos = {}
```

```
   go_col = re.split(r'\^|W|`',arr[9])
   # go over the GO terms one at a time and print the results
   # to the second output file
   for g in go_col:
      if not re.search("^GO",g):continue
      out2.write(gene+"\t"+g+"\n")

trino_file.close()
out1.close()
out2.close()
```

helper_methods.py

```
import re

# This methods receives a path of a mapping file
# which is a table in which column ind1 holds the keys
# and column ind2 holds the values
# key_sep specifices how to get the key values: none = do not split
def readMappingFile(path,sep1="\s+;\s+",ind1=0,ind2=1,key_sep=None,key_sep_ind=0):
   f = open (path,"r")
   d = {}
   for l in f:
      l = l.rstrip()
      arr = re.split(sep1,l)
      if len(arr)<ind2+1:continue
      k = arr[ind1]
      if key_sep!=None:
         #print re.split(key_sep,k)
         k = re.split(key_sep,k)[key_sep_ind]
      v = arr[ind2]
      # print k,v
      curr_list = d.get(k,[])
      curr_list.append(v)
      d[k]=curr_list
   f.close()
   return d
```

Supplemental Method 4. Preprocessing of gene expression data

We collected potato expression profiles from over 20 studies covering 326 experimental conditions. The raw data contained 52,998 probes. We applied quantile normalization using the Limma package (Smyth, 2005) and subtracted the background intensity from the foreground intensity for each spot using the normexp method (Ritchie et al., 2007).

We removed probes whose expression level was consistently low over the experimental conditions and thus reduce statistical noise when performing co-expression analysis (Tzfadia et al., 2012). We set an intensity level threshold of 204 based on the histogram curve of normalized intensities (see Fig. 2)., and removed probes whose expression was lower than the threshold in all experiments.

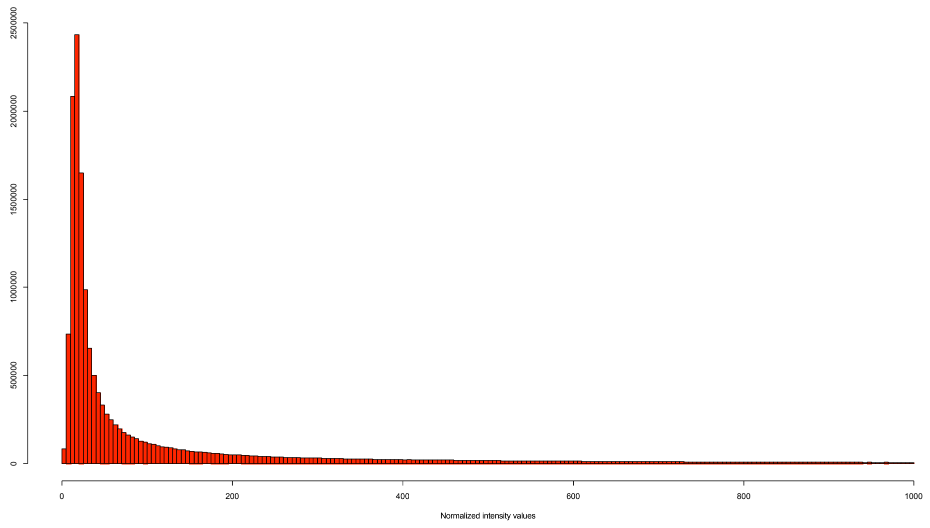

Fig. 2. Distribution of the normalized intensity values

In addition, we removed probes with low expression variance. The probe standard deviation distribution is shown below.

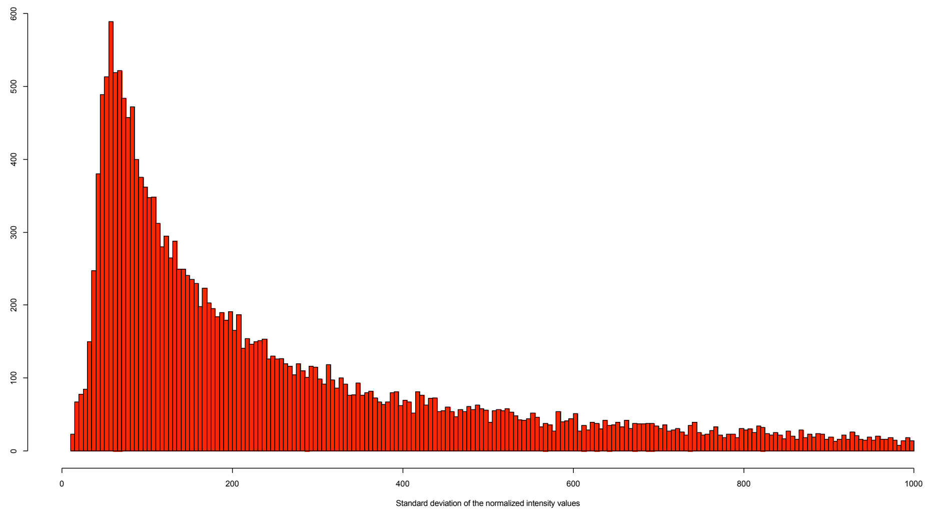

Fig. 3. Distribution of the standard deviation of the normalized intensity values across the different experimental conditions.

By setting a standard deviation of 312, we reduced the number of probes from 24,434 to 14,000 (see Fig. 3). These probes were mapped to 12,956 genes, approximately the same amount of genes analyzed in Tzfadia et al. (2012).

Supplemental Method 5. How to use the R scripts

This document describes the scripts for comparing pipelines, merging pipelines, and validations using gene expression data and a list of gold standard annotations. All scripts were written in R (2.15.1) and were tested both in Windows and UNIX.

1 REQUIREMENTS

The calculations of our tools could be computationally demanding. For example, to get GO-based similarity measures between pipelines we calculate semantic similarities between all GO terms that are present in the flows. For example, in the potato data we calculated similarities among >5000 GO biological process terms using the GOSemSim R package. As a rule of thumb, we recommend to use a machine with at least 8GB RAM memory. To run the scripts the following R packages should be installed: hash, GO.db, GOSemSim, corrplot, and multicore (optional, required only for running in Unix).

2 INPUT

To run our analyses the user should create a directory and put all data in three sub-directories:

- `flows` — output of the pipelines. For each pipeline add a tab-delimited file with two columns: gene id and GO term id.
- `gs` — gold standards. For each gold standard add a tab-delimited file with two columns: gene id and GO term id.
- `ge_data` — gene expression datasets. Each gene expression dataset is a tab-delimited text file in which columns are conditions and rows are genes. The first row contains the condition names (i.e., the header of the table) and the first column contains the gene names.

3 R FILES

- `flow_comp_methods.R`: all auxiliary functions. All data loading processes, measurements, and algorithms are implemented in this script.
- `MAIN.R`: the main script that runs the analysis. This script receives as input the name of the folder that contains all the data (divided to three sub-directories as explained above) and runs the following stages:

  a. Read the flows and remove duplications (genes and GO terms).
  b. Split the flows by GO types: MF, BP, and CC.
  c. Get GO-free similarity scores between the pipelines.
  d. Get GO-based similarity scores between the pipelines.
  e. Get the gold standard validation scores for each flow (GO-based and GO-free).
  f. Get the gene expression validation scores for each flow.
  g. Calculate the ensemble solutions of the pipelines.
  h. Validate the ensembles (gold standard and gene expression).
  i. Save all the results in RData files (see details in Output R objects). These files will be saved in a new directory called `output_objects`.

- `generate_plots_from_robjects.R`: a script that reads the results of the main script (i.e., the RData files) and can be used to generate plots that appear in the paper. See the comments within the scripts for details.

4 OUTPUT R OBJECTS

All results of the main scripts are saved in a new directory called `output_objects`. The list of objects can be seen in the table below.

Name	Type	Description/remarks
flows_as_mats	List*	For each pipeline keep a matrix with two columns. Each row: gene, GO term (no duplications)
flows_as_hashes	List*	For each pipeline a hash table is kept. Each hash table maps genes to their GO terms
flow2numannots	Vector*	For each pipeline: the number of annotations
flow2numgenes	Vector*	For each pipeline: the number of covered genes
flow2numGOsperGene	Vector*	For each pipeline: the average number of GO terms per gene
flow_preds	List*	For each pipeline a matrix of gene to GO annotation is kept (after removing duplications)
jaccard_matrix_annots	Matrix**	A matrix with the GO-free similarities between pipelines
flow_go_sims	List	A list of GO-based similarity matrices between flows. Currently implemented: BP and MF matrices.
YY_validation_gold_stand_XX	List	A list of gold standard validation results. Each list cell corresponds to a gold standard file and restores for each flow the precision, recall, and F-measure. XX can be bp (biological process), mf (molecular function), or free (GO-free measure). YY can be empty and keeps the results of the original flows, unsupervised are the results of the ensemble.
YY_flows_ge_validation_results_XX	List	A list of gene expression validation results. Each list cell corresponds to one gene expression dataset and restores for each flow the analysis results of all GO terms. XX can be meancorr (mean correlation), or pvals (p-values). YY can be empty and keeps the results of the original flows, unsupervised the results of the ensemble.
unsupervised_k2ensemble_as_mats	List	Similar to flows_as_mats but keeps the results of the ensemble method
unsupervised_k2ensemble_as_hashes	List	Similar to flows_as_hashes but keeps the results of the ensemble method

(*names are pipelines; **column and row names are pipelines)

5 SCRIPTS

flow_comp_methods.R

```
##### METHODS #####
# Author; David Amar - davidama@post.tau.ac.il
# Hackathon 2013, Amsterdam
################

######################################## Utilities to read the flows
    #############################

# This method reads the output file and and
# transform the predictions to characters
# so that we can perform set operations
# "#####" is used to separate between the gene id and
# the function id
# returns a vector of strings with the predictions
readFlowOutput <- function(f, show=T){
      if (show){print (f)}
      preds = read.table(f, sep="\t", header=F, stringsAsFactors=F)
      preds_ch = paste(preds[, 1], preds[, 2], sep="#####")
      return (preds_ch)
}

getFlowGenes<-function(m){
      return (unique(m[, 1]))
}

getFlowGOs<-function(m){
      return (unique(m[, 2]))
}
```

```
getFlowPreds <-function(preds){
      preds_ch = paste(preds[, 1], preds[, 2], sep="#####")
      return (preds_ch)
}

readFlowOutputTable <- function(f, show=T){
      if (show){print (f)}
      preds = read.table(f, sep="\t", header=F, stringsAsFactors=F)
      preds = (as.matrix(preds))
      mode(preds) = "character"
      to_r = which(apply(preds=="", 1, any))
      if (length(to_r)>0){preds = preds[-to_r, ]}
      return (preds)
}


getValueSetInColumn<-function(f, column){
      preds = read.table(f, sep="\t", header=F)
      return (unique(preds[, column]))
}

getGO2genesHash<-function(mat, as_hash=T, mfancs=NULL, bpancs=NULL, ccancs=NULL){
      if(is.null(dim(mat))){return (hash())}
      res = hash()
      if (!as_hash){res=list()}
      n = dim(mat)[1]
      for (i in 1:n){
            gene = mat[i, 1]; go = mat[i, 2]
            if (go=="" || gene == ""){next}
            gos=c(go)
            if (!is.null(mfancs)){gos = c(gos, mfancs[[go]])}
            if (!is.null(bpancs)){gos = c(gos, bpancs[[go]])}
            if (!is.null(ccancs)){gos = c(gos, ccancs[[go]])}
            for (currgo in gos){
                  if (as_hash && !has.key(key=currgo, res)){
                        res[[currgo]] = c(gene)
                        next
                  }
                  curr_genes = res[[currgo]]
                  curr_genes = unique(c(curr_genes, gene))
                  res[[currgo]] = curr_genes
            }
      }
      return (res)
}

getgene2GOHash<-function(mat, as_hash=T){
      if(is.null(dim(mat))){return (hash())}
      res = hash()
      if (!as_hash){res=list()}
      n = dim(mat)[1]
      for (i in 1:n){
            gene = mat[i, 1]; go = mat[i, 2]
            if (go=="" || gene == ""){next}
            if (as_hash && !has.key(key=gene, res)){
                  res[[gene]] = c(go)
                  next
            }
            curr_gos = res[[gene]]
            curr_gos = unique(c(curr_gos, go))
            res[[gene]] = curr_gos
      }
      return (res)
}

#getFileNameToToolName<-function(files){
#     file_program_name = c()
#     for (f in files){
#           if (grepl(pattern = "tri_pfam", f,
    ignore.case=T)){file_program_name[f]="Tri_HMM";next}
```

```
#           if (grepl(pattern = "tri_GO", f,
    ignore.case=T)){file_program_name[f]="Tri_BLAST";next}
#           if (grepl(pattern = "trinotate_pfam2go", f,
    ignore.case=T)){file_program_name[f]="Tri_HMM";next}
#           if (grepl(pattern = "trinotate_directGO", f,
    ignore.case=T)){file_program_name[f]="Tri_BLAST";next}
#           if (grepl(pattern = "Phytozome", f,
    ignore.case=T)){file_program_name[f]="Phytozome";next}
#           if (grepl(pattern = "B2G", f, ignore.case=T) && grepl(pattern = "swissprot", f,
    ignore.case=T)){file_program_name[f]="BLAST2GO_sp";next}
#           if (grepl(pattern = "B2GO", f, ignore.case=T)&& grepl(pattern = "swissprot", f,
    ignore.case=T)){file_program_name[f]="BLAST2GO_sp";next}
#           if (grepl(pattern = "BLAST2GO", f, ignore.case=T)&& grepl(pattern = "swissprot", f,
    ignore.case=T)){file_program_name[f]="BLAST2GO_sp";next}
#           if (grepl(pattern = "B2G", f, ignore.case=T)){file_program_name[f]="BLAST2GO";next}
#           if (grepl(pattern = "B2GO", f, ignore.case=T)){file_program_name[f]="BLAST2GO";next}
#           if (grepl(pattern = "BLAST2GO", f,
    ignore.case=T)){file_program_name[f]="BLAST2GO";next}
#           if (grepl(pattern = "PotatoCyc", f,
    ignore.case=T)){file_program_name[f]="PotatoCyc";next}
#           if (grepl(pattern = "METASTUDENT", f,
    ignore.case=T)){file_program_name[f]="Metastudent";next}
#           if (grepl(pattern = "OrthoMCL", f, ignore.case=T)&& grepl(pattern = "swissprot", f,
    ignore.case=T)){file_program_name[f]="OrthoMCL_sp";next}
#           if (grepl(pattern = "OrthoMCL", f,
    ignore.case=T)){file_program_name[f]="OrthoMCL_nr";next}
#           if (grepl(pattern = "biomart", f,
    ignore.case=T)){file_program_name[f]="BioMart";next}
#     }
#     return (file_program_name)
#}

######################################### Utilities for flow comparison and validation
    ############################

getJaccardScoreGeneIntersect<-function(preds1, preds2){
      genes1 = unique(preds1[, 1]);genes2=unique(preds2[, 1])
      genes = intersect(genes1, genes2)
      inGenes1 = which(sapply(preds1[, 1], is.element, set=genes))
      inGenes2 = which(sapply(preds2[, 1], is.element, set=genes))
      preds1_ch = paste(preds1[inGenes1, 1], preds1[inGenes1, 2], sep="#####")
      preds2_ch = paste(preds2[inGenes2, 1], preds2[inGenes2, 2], sep="#####")
      return(getJaccardScore(preds1_ch, preds2_ch))
}


getJaccardScore<-function(preds1_ch, preds2_ch){
      pred1_pred2_intersect = intersect(preds1_ch, preds2_ch)
      pred1_pred2_union = union(preds1_ch, preds2_ch)
      jaccard = length(pred1_pred2_intersect)/length(pred1_pred2_union)
      return(jaccard)
}

getJaccardMatrix<-function(flow_preds, func = getJaccardScore){
      files = names(flow_preds)
      n = length(files)
      j_matrix = matrix(ncol=n, nrow=n);diag(j_matrix)=1
      rownames(j_matrix)<-files; colnames(j_matrix)<-files
      for (i in 2:n){
            for (j in 1:(i-1)){
                  j_matrix[i, j] = func(flow_preds[[i]], flow_preds[[j]])
                  j_matrix[j, i] = j_matrix[i, j]
            }
      }
      return(j_matrix)
}

# calculate precision, recall, and f-measure
# preds2 is the gold standard
getAccuracyMeasures<-function(preds1, preds2){
```

```r
	if (length(preds1)==0 || length(preds2)==0){return(list(precision=0, recall=0, F=0))}
	if (is.null(dim(preds1))){preds1=matrix(preds1, nrow=1)}
	if (is.null(dim(preds2))){preds1=matrix(preds2, nrow=1)}
	preds1_ch = paste(preds1[, 1], preds1[, 2], sep="#####")
	preds2_ch = paste(preds2[, 1], preds2[, 2], sep="#####")
	f2_genes = unique(as.character(preds2[, 1]))
	f2_genes_hash = hash()
	for (g in f2_genes){f2_genes_hash[[g]]=1}
	rows_in_preds1_with_preds2_genes = which(sapply(as.character(preds1[, 1]), hash::has.key,
	    hash=f2_genes_hash))
	if (length(rows_in_preds1_with_preds2_genes)==0){return(list(precision=0, recall=0, F=0))}
	curr_preds1 = matrix(preds1[rows_in_preds1_with_preds2_genes, ],
	    nrow=length(rows_in_preds1_with_preds2_genes))
	curr_preds1_ch = paste(curr_preds1[, 1], curr_preds1[, 2], sep="#####")
	TP = length(intersect(preds2_ch, curr_preds1_ch))
	FP = length(setdiff(curr_preds1_ch, preds2_ch))
	FN = length(setdiff(preds2_ch, curr_preds1_ch))
	precision=0
	if((TP+FP)>0){precision = TP/(TP+FP)}
	recall=0
	if((TP+FN)>0){recall = TP/(TP+FN)}
	F_measure=0
	if ((precision+recall)>0){F_measure = 2*((precision*recall)/(precision+recall))}
	results = list(precision=precision, recall=recall, F=F_measure)
	return(results)
}

# go based accuracy
# gs and preds2 are hash tables mapping
# genes to their GO terms
# this method uses only the go terms
# in the rownames of sims
getGOBasedAccuracyMeasures<-function(preds, gs, sims){
	gs_genes = hash::keys(gs)
	precision = 0
	recall = 0
	count_annotated_gs_genes = 0
	count_annotated_pred_genes = 0
	for (gene in gs_genes){
		if (!hash::has.key(gene, hash=preds)){next}
		gs_curr_annots = intersect(rownames(sims), gs[[gene]])
		preds_curr_annots = intersect(rownames(sims), preds[[gene]])
		if (length(gs_curr_annots)>0){count_annotated_gs_genes=count_annotated_gs_genes+1}
		if
		    (length(preds_curr_annots)>0){count_annotated_pred_genes=count_annotated_pred_genes+1}
		if (length(gs_curr_annots)==0||length(preds_curr_annots)==0){next}
		currmat = sims[gs_curr_annots, preds_curr_annots]
		currmat = matrix(currmat, ncol=length(gs_curr_annots),
		    nrow=length(preds_curr_annots))
		curr_prec = mean(apply(currmat, 1, max))
		curr_recall = mean(apply(currmat, 2, max))
		precision = precision+curr_prec
		recall = recall + curr_recall
	}
	precision = precision / length(gs_genes)
	recall = recall / length(gs_genes)
	F_measure=0
	if ((precision+recall)>0){F_measure = 2*((precision*recall)/(precision+recall))}
	results = list(precision=precision, recall=recall, F=F_measure)
	return(results)
}

getFlowsPerformance<-function(flows, performance_function, gs_files, gs_flows, useMC=F,
    numCores=1, ...){
	results = list()
	for (gsname in gs_files){
		GS = gs_flows[[gsname]]
		if (useMC){
			validation_scores = mclapply(flows, performance_function, GS, ...,
			    mc.cores=numCores)
```

```
                names(validation_scores) = names(flows)
                validation_scores = as.matrix(sapply(validation_scores, unlist))
            }
            else{
                validation_scores = sapply(flows, performance_function, GS, ...)
            }
            results[[gsname]] = validation_scores
        }
        return (results)
}



################################ calculate statistics of a single flow
    ####################################
####### Based on ideas from Defoin-Paltel et al. BMC bioinfo 2011

# x is a matrix in which each row has
# a gene and a go term
getFlowNumGenes<-function(x){
        return (unique(x[, 1]))
}

# x is a matrix in which each row has
# a gene and a go term
getFlowNumGOs<-function(x){
        return (unique(x[, 2]))
}

# x is a matrix in which each row has
# a gene and a go term
getFlowMeanNumGOsPerGene<-function(x){
        return (dim(x)[1]/length(getFlowNumGenes(x)))
}

################################# Working with GO.db and GO similarities
    ####################################
library(GO.db)
getGO2AnnotationType<-function(additional_info_file = "../go_raw_data_analysis/go2ont.txt"){
        allgos = c(
          names(as.list(GOCCCHILDREN)),
          names(as.list(GOBPCHILDREN)),
          names(as.list(GOMFCHILDREN))
          )
        allannots = c(
          rep("CC", length(as.list(GOCCCHILDREN))),
          rep("BP", length(as.list(GOBPCHILDREN))),
          rep("MF", length(as.list(GOMFCHILDREN)))
        )
        names(allannots)<-allgos

        # read the mapping from a file
        # this file contains the mapping taken from
        # the gene ontology raw data
        if (!is.null(additional_info_file )){
               go2t = read.table(additional_info_file, header=F, row.names=1)
               go2type = as.character(go2t[, 1]); names(go2type)=rownames(go2t)
               go2type[which(go2type=="biological_process")]="BP"
               go2type[which(go2type=="molecular_function")]="MF"
               go2type[which(go2type=="cellular_component")]="CC"
        }

        # merge both mapping files
        gonames = unique(c(names(allannots), names(go2type)))
        go2ontologyType = c()
        go2ontologyType[names(allannots)]=allannots
        if (!is.null(additional_info_file )){
              go2ontologyType[names(go2type)]=go2type
        }
        return (go2ontologyType)
```

```
}

getMostSpecificGOs<-function(termslist, bplist, cclist, mflist){
	if (length(termslist)==0){return (c())}
	for (currgo in termslist){
		termslist = removeAncestorsFromSet(currgo, bplist, termslist)
		termslist = removeAncestorsFromSet(currgo, cclist, termslist)
		termslist = removeAncestorsFromSet(currgo, mflist, termslist)
	}
	return (termslist)
}

removeAncestorsFromSet<-function(go, ancslist, s){
	return (setdiff(s, ancslist[[go]]))
}

removeDuplicationsFromAnnotations<-function(gene2gos, bplist, cclist, mflist){
	newGene2Go = hash()
	newAsMat = c()
	genes = hash::keys(gene2gos)
	total_removed = 0
	for (gene in genes){
		currList = gene2gos[[gene]]
		before = length(currList)
		currList = getMostSpecificGOs(currList, bplist, cclist, mflist)
		after = length(currList)
		total_removed = total_removed + (before-after)
		newGene2Go[[gene]] = currList
		curr_as_matrix = c()
		for (go in currList){
			curr_as_matrix= rbind(curr_as_matrix, c(gene, go))
		}
		newAsMat = rbind(newAsMat, curr_as_matrix)
	}
	return (list(newGene2Go=newGene2Go, newAsMat=newAsMat, total_removed=total_removed))
}

getGOTerms<-function(type, files, path=NULL, flow_mats=NULL){
	if (!is.null(path)){files=paste(path, files, sep="/")}
	goterms = c()
	for (f in files){
		if (! grepl(pattern = type, f)){next}
		if (is.null(flow_mats)){
			t = readFlowOutputTable(f)
		}
		else{
			t = flow_mats[[f]]
		}
		gos = as.character(t[, 2])
		goterms = unique(c(goterms, gos))
	}
	return (goterms)
}


getSimMat<-function(type, goterms){
	n = length(goterms)
	sims_mat = matrix(rep(0, size=n*n), ncol=n, nrow=n); diag(sims_mat) = 1
	for (i in 2:n){
		for (j in 1:(i-1)){
			sims_mat[i, j]=goSim(goterms[i], goterms[j], ont=type, measure="Wang")
		}
	}
	for (i in 2:n){
		for (j in 1:(i-1)){
			sims_mat[j, i] = sims_mat[i, j]
		}
	}
	is_na2 = is.na(sims_mat);sims_mat[is_na2]=0
	rownames(sims_mat)=goterms; colnames(sims_mat)=goterms
```

```
    return (sims_mat)
}

# given two sets of go terms, this methods calculates a summary
# score based on the running max average
getRunMaxAvgSim<-function(gos1, gos2, sims){
    m = sims[gos1, gos2]
    if (length(gos1)==1 || length(gos2)==1){
        return (mean(m))
    }
    avsb = apply(m, 1, max)
    bvsa = apply(m, 2, max)
    mn = length(avsb)+length(bvsa)
    score = (sum(avsb)+sum(bvsa))/mn
    #print (paste("##", score))
    return(score)
}

# get the mean similarity of a set of go terms
# good for comparing the similarity of annotations
# of a gene (e.g., within a single flow)
getMeanSim<-function(gos, sims){
    return (mean(sims[gos, gos]))
}

# this method calculates the semantic similarity between two flows
compareTwoFlowsUsingSimMat<-function(h1, h2, genes=NULL, sims){
    if (is.null(genes)){
        genes = unique(c(hash::keys(h1), hash::keys(h2)))
    }
    scores = sapply(genes, getGeneSimScore, h1=h1, h2=h2, sims=sims)
    return (mean(scores))
}

# this method calculates the similarity between
# the predicted gGO terms of a specific gene
getGeneSimScore<-function(g, h1, h2, sims){
    s1 = h1[[g]];s1=intersect(s1, rownames(sims))
    s2 = h2[[g]];s2=intersect(s2, rownames(sims))
    if (is.null(s1) || is.null(s2)|| length(s1)==0 || length(s2)==0){return (0)}
    return (getRunMaxAvgSim(s1, s2, sims))
}

# this is the wrapper method that
# calculates all pairwise similarities between the flows
getSemSimMatrix<-function(flow_preds, sims){
    files = names(flow_preds)
    n = length(files)
    j_matrix = matrix(ncol=n, nrow=n);diag(j_matrix)=1
    rownames(j_matrix)<-files; colnames(j_matrix)<-files
    for (i in 2:n){
        for (j in 1:(i-1)){
            j_matrix[i, j] = compareTwoFlowsUsingSimMat(flow_preds[[i]], flow_preds[[j]],
                sims=sims)
            j_matrix[j, i] = j_matrix[i, j]
        }
    }
    return(j_matrix)
}

################################# Gene expression analysis methods ############################

getMeanCoexpOfGO <- function(goid, flow_h, corr_mat, minSize=2, maxSize=500){
    # this is a simple yet slow way to get the genes of a GO term directly from the
    # flow prediction matrix, we used it for QA to make sure that our hash tables
    # are correct curr_row_inds = which(mat[, 2]==goid)
    curr_genes = flow_h[[goid]]
    if (is.null(curr_genes) || length(curr_genes)<minSize ||
        length(curr_genes)>maxSize){return(NA)}
    m = corr_mat[curr_genes, curr_genes]
```

```
	lt = m[lower.tri(m)]
	return (mean(lt))
}

getCoexpOfGOPvalue <- function(goid, flow_h, corr_mat, bg_corrs_vector, minSize=2, maxSize=500){
	curr_genes = flow_h[[goid]]
	if (is.null(curr_genes) || length(curr_genes)<minSize ||
	    length(curr_genes)>maxSize){return(NA)}
	m = corr_mat[curr_genes, curr_genes]
	lt = m[lower.tri(m)]
	return (ks.test(bg_corrs_vector, lt, alternative="greater")$p.value)
}

getCoexpOfGOPvalueSamplingBased <- function(goid, flow_h, corr_mat, minSize=2, maxSize=500,
    n=50, repeats=50, ...){
	curr_genes = flow_h[[goid]]
	if (is.null(curr_genes) || length(curr_genes)<minSize ||
	    length(curr_genes)>maxSize){return(NA)}
	m = corr_mat[curr_genes, curr_genes]
	pvals = c()
	for (j in 1:repeats){
		randCorrs = getRandCorrelarions(corr_mat, n=n)
		realCorrs = getRandCorrelarions(m, n=min(n, length(curr_genes)))
		pvals[j] = getPval(randCorrs, realCorrs, ...)
	}
	return (mean(pvals))
}

getPval<-function(x, y, alternative="gr", test=wilcox.test, ...){
    return (test(y, x, alternative=alternative, ...)$p.value)
}

getSimMatStatistics<-function(m, f=mean){
	m = corr_mat[curr_genes, curr_genes]
	lt = m[lower.tri(m)]
	return (f(lt))
}

getRandCorrelarions<-function(corrs_mat, n, asVector=T){
	shuff = sample(1:dim(corrs_mat)[1])
	n = min(n, dim(corrs_mat)[1])
	if (n==0){return (c())}
	inds = shuff[1:n]
	m = corrs_mat[inds, inds]
	if (asVector){
		lt = m[lower.tri(m)]
		return (lt)
	}
	return (m)
}

removePredictionsByBG<-function(mat, bg){
	is_in_bg = which(sapply(mat[, 1], hash::has.key, hash=bg))
	return (mat[is_in_bg, ])
}

# parameters:
# curr_mats = a list of strings that represent the paths of the gene expression matrices
# goterms: a list of go terms that shall be tested by the function
run_gene_expression_validation<-function(flows_as_mats, curr_mats, goterms,
    max_go_term_size=500, min_go_term_size=3, useMC=F, numCores=1){
	tmp_ks = list()
	tmp_meancorr = list()
	bpancs=as.list(GOBPANCESTOR);mfancs=as.list(GOMFANCESTOR);ccancs=as.list(GOCCANCESTOR)
	for (mat_path in curr_mats){
		runname = mat_path
		data = read.table(mat_path, sep="\t", row.names=1, header=T)
		data = as.matrix(data)
		if (max(data)>100){data = log(data)}
		corrs_mat = cor(t(data))
```

```
		genes_in_exp_mat = rownames(corrs_mat)
		bg = hash();for (gene in genes_in_exp_mat){bg[[gene]]=1}
		flows = names(flows_as_mats)
		t_flow_mats = sapply(flows_as_mats, removePredictionsByBG, bg=bg)
		t_flow_mats_h = sapply(t_flow_mats, getGO2genesHash, mfancs=mfancs, bpancs=bpancs,
		    ccancs=ccancs)
		print ("finished mapping GOs to their genes")
		go_flow_mean_matrix = matrix(nrow=length(goterms), ncol=length(flows))
		rownames(go_flow_mean_matrix) = goterms;colnames(go_flow_mean_matrix) = flows
		for (f in flows){
			if (useMC){
				go_flow_mean_matrix[, f] = unlist(mclapply(goterms, getMeanCoexpOfGO,
				    flow_h = t_flow_mats_h[[f]],
							corr_mat=corrs_mat, minSize=min_go_term_size,
							    maxSize=max_go_term_size, mc.cores=numCores))
			}
			else{
				go_flow_mean_matrix[, f] = sapply(goterms, getMeanCoexpOfGO, flow_h =
				    t_flow_mats_h[[f]],
							corr_mat=corrs_mat, minSize=min_go_term_size,
							    maxSize=max_go_term_size)
			}
		}
		tmp_meancorr[[runname]] = go_flow_mean_matrix
		print (paste("mean correlation of GO terms: ", mean(go_flow_mean_matrix, na.rm=T)))
		go_flow_kspval_matrix = matrix(nrow=length(goterms), ncol=length(flows))
		rownames(go_flow_kspval_matrix) = goterms;colnames(go_flow_kspval_matrix) = flows
		for (f in flows){
			if (useMC){
				go_flow_kspval_matrix[, f]= unlist(mclapply(goterms,
				    getCoexpOfGOPvalueSamplingBased, flow_h = t_flow_mats_h[[f]],
							corr_mat=corrs_mat, minSize=min_go_term_size,
							    maxSize=max_go_term_size, test=t.test,
							    mc.cores=numCores))
			}
			else{
				go_flow_kspval_matrix[, f]= sapply(goterms,
				    getCoexpOfGOPvalueSamplingBased, flow_h = t_flow_mats_h[[f]],
							corr_mat=corrs_mat, minSize=min_go_term_size,
							    maxSize=max_go_term_size, test=t.test)
			}
		}
		tmp_ks[[runname]] = go_flow_kspval_matrix
		print (paste("mean p-value of GO terms: ", mean(go_flow_kspval_matrix, na.rm=T)))
		gc()
	}
	return (list(pval_results = tmp_ks, meancorr_results = tmp_meancorr))
}

############################################## The simple ensemble: implementation
    ####################################################
library(GO.db)
fromPredListToMatrix<-function(preds, ancslist, genes, goterms){
	n = length(genes);m=length(goterms)
	m = matrix(rep(0, times=n*m), nrow=n, ncol=m)
	rownames(m)=genes;colnames(m)=goterms
	for (i in 1:dim(preds)[1]){
		gene=preds[i, 1];go=preds[i, 2]
		if (!is.element(gene, set=genes)){next}
		if (!is.element(go, set=goterms)){next}
		m[gene, go]=1
		if (!is.element(go, set=names(ancslist))){next}
		curr_ancs = intersect(ancslist[[go]], goterms)
		if (length(curr_ancs)>0){m[gene, curr_ancs]=1}
	}
	return (m)
}

getSumOfMats<-function(flow_mats, ancslist){
	# get all go terms and all genes
```

```
	goterms = c();genes=c()
	for (f in flow_mats){
		goterms = c(goterms, unique(f[, 2]))
		genes = c(genes, unique(f[, 1]))
	}
	genes = unique(genes)
	goterms = intersect(unique(goterms), names(ancslist))
	n = length(genes);m=length(goterms)
	m = matrix(rep(0, times=n*m), nrow=n, ncol=m)
	rownames(m)=genes;colnames(m)=goterms
	for (f in flow_mats){
		print (mean(m))
		m = m + fromPredListToMatrix(f, ancslist, genes, goterms)
		gc()
	}
	return(m)
}

extractAnnotations<-function(m, k, ancslist){
	annots = apply(m, 1, getMostSpecificTerms, k=k, gonames=colnames(m), ancslist=ancslist)
	return (annots)
}

getMostSpecificTerms<-function(x, k, gonames, ancslist){
	termslist = gonames[which(x>=k)]
	if (length(termslist)==0){return (c())}
	for (currgo in termslist){
		termslist = removeAncestorsFromSet(currgo, ancslist, termslist)
	}
	return (termslist)
}


predListToMatix<-function(p){
	mat=c()
	for (gene in names(p)){
		golist=p[[gene]]
		for (go in golist){
			mat = rbind(mat, c(gene, go))
		}
	}
	return (mat)
}

getAllKEnsembleOfFlows<-function(curr_files, flows_as_mats, ancslists, maxK=NULL){
	k2ensemble=list()
	sum_of_annots_bp = getSumOfMats(flows_as_mats[curr_files], ancslists)
	if (is.null(maxK)){maxK=length(curr_files)}
	for (k in 1:maxK){
		annots = extractAnnotations(sum_of_annots_bp, k, ancslists)
		annots_as_mat = predListToMatix(annots)

		currkey = as.character(k)
		currlist = k2ensemble[[currkey]]
		currlist = rbind(currlist, annots_as_mat)
		k2ensemble[[currkey]] = currlist
	}
	sum_of_annots_bp = NULL
	gc()
	return (k2ensemble)
}
```

---

generate_plots_from_robjects.R

```
# IMPORTANT: Read the comments below on how to set the needed directories and files

# Set the path in which the robjects were saved.
# The robjects are created by the MAIN.R script
```

```
PATH = ""
setwd(PATH)

# Plot parameters
library(corrplot)
margins = c(15, 5, 5, 5)
textcex=1.7
las=2
barplot_space = c(0, 0.3)

# Set the directory in which the R scripts are saved
scripts_dir = ""
source(paste(scripts_dir, "flow_comp_methods.R", sep="/"))

############# Auxiliary method for flow names ################
# If you use additional\other pipelines, set the names you want in the
# plots here
getFileNameToToolName<-function(files){
      file_program_name = c()
      for (f in files){
            if (grepl(pattern = "tri_pfam", f,
                ignore.case=T)){file_program_name[f]="Tri_HMM";next}
            if (grepl(pattern = "tri_GO", f,
                ignore.case=T)){file_program_name[f]="Tri_BLAST";next}
            if (grepl(pattern = "trinotate_pfam2go", f,
                ignore.case=T)){file_program_name[f]="Tri_HMM";next}
            if (grepl(pattern = "trinotate_directGO", f,
                ignore.case=T)){file_program_name[f]="Tri_BLAST";next}
            if (grepl(pattern = "Phytozome", f,
                ignore.case=T)){file_program_name[f]="Phytozome";next}
            if (grepl(pattern = "B2G", f, ignore.case=T)){file_program_name[f]="BLAST2GO";next}
            if (grepl(pattern = "B2GO", f, ignore.case=T)){file_program_name[f]="BLAST2GO";next}
            if (grepl(pattern = "BLAST2GO", f,
                ignore.case=T)){file_program_name[f]="BLAST2GO";next}
            if (grepl(pattern = "OrthoMCL", f,
                ignore.case=T)){file_program_name[f]="OrthoMCL";next}
            if (grepl(pattern = "biomart", f,
                ignore.case=T)){file_program_name[f]="BioMart";next}
      }
      return (file_program_name)
}
###################################################################

########################### plot the annotations jaccard matrix #####################
load("flows_as_mats.RData")
load("jaccard_matrix_annots.RData")
files = names(flows_as_mats)
tl.cex = 1.7

splitted_flows = which(grepl(files, pattern = "_(BP)|(CC)|(MF)", perl=T, ignore.case=T))
unsplitted_flows = files[-splitted_flows]
x = jaccard_matrix_annots[unsplitted_flows, unsplitted_flows]
mean(x[lower.tri(x)])
rownames(x) = getFileNameToToolName(unsplitted_flows);colnames(x) =
    getFileNameToToolName(unsplitted_flows)
col3 <- colorRampPalette(c("white", "black"))
corrplot(x, is.corr=F, order="hclust", main="GO-free comparison using the Jaccard score",
    mar=c(2, 0, 2, 0), tl.cex=tl.cex, tl.col="black")

BP_inds = which(grepl(colnames(jaccard_matrix_annots), pattern = '_BP.txt'))
x = jaccard_matrix_annots[BP_inds, BP_inds]
rownames(x) = getFileNameToToolName(colnames(jaccard_matrix_annots)[BP_inds]);colnames(x) =
    getFileNameToToolName(colnames(jaccard_matrix_annots)[BP_inds])
corrplot(x, is.corr=F, order="hclust", main="GO BP-free comparison using the Jaccard score",
    mar=c(2, 0, 2, 0), cl.lim=c(0, 1), tl.cex=tl.cex, tl.col="black")

MF_inds = which(grepl(colnames(jaccard_matrix_annots), pattern = '_MF.txt'))
x = jaccard_matrix_annots[MF_inds, MF_inds]
rownames(x) = getFileNameToToolName(colnames(jaccard_matrix_annots)[MF_inds]);colnames(x) =
    getFileNameToToolName(colnames(jaccard_matrix_annots)[MF_inds])
```

```
corrplot(x, is.corr=F, order="hclust", main="GO MF-free comparison using the Jaccard score",
    mar=c(2, 0, 2, 0), cl.lim=c(0, 1), tl.cex=tl.cex, tl.col="black")
mean(x[lower.tri(x)])

# genes jaccard
getGenes<-function(flow){return (unique(flow[, 1]))}
flow_genes = sapply(flows_as_mats[unsplitted_flows], getGenes)
jaccard_matrix_genes = getJaccardMatrix(flow_genes)
x = jaccard_matrix_genes
rownames(x) = getFileNameToToolName(colnames(jaccard_matrix_genes));colnames(x) =
    getFileNameToToolName(colnames(jaccard_matrix_genes))
corrplot(x, is.corr=F, order="hclust", main="Gene set comparison using the Jaccard score",
    mar=c(2, 0, 2, 0), cl.lim=c(0, 1), tl.cex=tl.cex, tl.col="black")
mean(x[lower.tri(x)])

# jaccard of the predictions using the gene intersection
preds_jaccard_gene_intersect = getJaccardMatrix(flows_as_mats[unsplitted_flows],
    getJaccardScoreGeneIntersect)
mean(preds_jaccard_gene_intersect[lower.tri(preds_jaccard_gene_intersect)])


########################### Display item 1 A: GO-based similarity
    ########################################
load("flow_go_sims.RData")
#par(mfrow=c(1, 2))
x = flow_go_sims[[1]]
mean(x[lower.tri(x)])
rownames(x) = getFileNameToToolName(rownames(x))
colnames(x) = getFileNameToToolName(colnames(x))
corrplot(x, is.corr=F, order="hclust", main="GO BP similarity", mar=c(2, 0, 2, 0), cl.lim=c(0,
    1), tl.cex=tl.cex, tl.col="black")
x = flow_go_sims[[2]]
mean(x[lower.tri(x)])
rownames(x) = getFileNameToToolName(rownames(x))
colnames(x) = getFileNameToToolName(colnames(x))
corrplot(x, is.corr=F, order="hclust", main="GO MF similarity", mar=c(2, 0, 2, 0), cl.lim=c(0,
    1), tl.cex=tl.cex, tl.col="black")

mean(flow_go_sims[[1]][lower.tri(flow_go_sims[[1]])])
mean(flow_go_sims[[2]][lower.tri(flow_go_sims[[2]])])

########################### Display item 1 B: flow statistics
    ########################################
load("flows_as_mats.RData")
load("unsupervised_k2ensemble_as_mats.RData")
files = names(flows_as_mats)
splitted_flows = which(grepl(files, pattern = "_(BP)|(CC)|(MF).txt", perl=T, ignore.case=T))
unsplitted_flow_files = files[-splitted_flows]
unsplitted_flows = flows_as_mats[unsplitted_flow_files]
names(unsplitted_flows) = getFileNameToToolName(unsplitted_flow_files)
all_flows = c(unsplitted_flows, unsupervised_k2ensemble_as_mats)
flow_genes = sapply(all_flows, getFlowGenes)
flow_gos = sapply(all_flows, getFlowGOs)
flow2numgenes = sapply(flow_genes, length)
par(mar=margins)
barplot(t(flow2numgenes), beside=T, main="Number of covered genes", cex.names=textcex, las=las,
    space=barplot_space)
flow2numgos = sapply(flow_gos, length)
flow2numannots = sapply(all_flows, length)/2
flow2numGOsPerGene = flow2numannots/flow2numgenes
par(mar=margins)
barplot(t(flow2numGOsPerGene), beside=T, main="Mean number of GO terms per gene",
    cex.names=textcex, las=las, space=barplot_space)

############################# Display item: gold standard validation
    #######################################
load("unsupervised_validation_gold_stand_go_free.RData")
load("unsupervised_validation_gold_stand_go_mf.RData")
load("unsupervised_validation_gold_stand_go_bp.RData")
load("validation_gold_stand_go_free.RData")
```

```
load("validation_gold_stand_go_mf.RData")
load("validation_gold_stand_go_bp.RData")
load("flows_as_mats.RData")
files = names(flows_as_mats)

# BP
bp_files = files[grepl(files, pattern="_BP.txt")]
x1 = validation_gold_stand_go_bp;x2=unsupervised_validation_gold_stand_go_bp
bp_gs = c()
inds = 2
for (ind in inds){
      bp_gs = rbind(bp_gs, c(x1[[ind]]["F", bp_files], x2[[ind]]["F", ]))
}
colnames(bp_gs) = c(getFileNameToToolName(bp_files), colnames(x2[[1]]))
rownames(bp_gs) = gsub(names(x1)[inds], pattern="(../gold_standards/)|(.txt)", replace="",
    perl=T)
mode(bp_gs) = "numeric"
pname = c("PotatoGold: BP")
for (i in 1:length(inds)){
      par(mar=margins)
      unsupervised_methods_bp = which(grepl(colnames(bp_gs), pattern="Unsup:k", ignore.case=F))
      n1 = length(colnames(bp_gs))-length(unsupervised_methods_bp)
      n2 = length(unsupervised_methods_bp)
      colnames(bp_gs) = gsub(colnames(bp_gs), pattern='Unsup:', replace='Ensemble:')
      barplot(bp_gs[i, ], beside=T, ylab="F-measure", ylim=c(0.3, 0.9), col=c(rainbow(n1),
          gray.colors(n2)), main=pname[i], xpd=F, cex.names=1.7, las=las, space=barplot_space,
          cex.lab=textcex)
}

# MF
mf_files = files[grepl(files, pattern="_MF.txt")]
x1 = validation_gold_stand_go_mf;x2=unsupervised_validation_gold_stand_go_mf
mf_gs = c()
inds = 1:length(x1)
for (ind in inds){
      mf_gs = rbind(mf_gs, c(x1[[ind]]["F", mf_files], x2[[ind]]["F", ]))
}
colnames(mf_gs) = c(getFileNameToToolName(mf_files), colnames(x2[[1]]))
rownames(mf_gs) = gsub(names(x1)[inds], pattern="(../gold_standards/)|(.txt)", replace="",
    perl=T)
mode(mf_gs) = "numeric"
pname = c("PotatoCyc", "PotatoGold")
#par(mfrow=c(1, length(inds)))
colnames(mf_gs) = gsub(colnames(mf_gs), pattern='Unsup:', replace='Ensemble:')
for (i in 2:length(inds)){
      par(mar=margins)
      unsupervised_methods_mf = which(grepl(colnames(mf_gs), pattern="Ensemble:k",
          ignore.case=F))
      n1 = length(colnames(mf_gs))-length(unsupervised_methods_mf)
      n2 = length(unsupervised_methods_mf)
      barplot(mf_gs[i, ], beside=T, ylab="F-measure", ylim=c(0.2, 0.9), col=c(rainbow(n1),
          gray.colors(n2)), main=pname[i], xpd=F, cex.names=1.7, las=las, space=barplot_space,
          cex.lab=textcex)
}

############################ Display item: gene expression validation
    ########################################
load ("unsupervised_flows_ge_validation_results_pvals.RData")
load ("unsupervised_flows_ge_validation_results_meancorr.RData")
load ("flows_ge_validation_results_pvals.RData")
load ("flows_ge_validation_results_meancorr.RData")

x1 = flows_ge_validation_results_pvals[[1]][,
    bp_files];x2=unsupervised_flows_ge_validation_results_pvals[[1]];
colnames(x1) = getFileNameToToolName(colnames(x1))
allgos = unique(c(rownames(x1), rownames(x2)))
allflows = c(colnames(x1), colnames(x2))
n=length(allgos); m = length(allflows)
xpvals = matrix(nrow=n, ncol=m)
rownames(xpvals) = allgos
```

```
xpvals[rownames(x1), 1:dim(x1)[2]] = x1
xpvals[rownames(x2), (1+dim(x1)[2]):(dim(x1)[2]+dim(x2)[2])] = x2
colnames(xpvals) = allflows

unsupervised_methods = which(grepl(colnames(xpvals), pattern="Unsup:k", ignore.case=F))
n1 = length(colnames(xpvals))-length(unsupervised_methods)
n2 = length(unsupervised_methods)

#par(mfrow=c(2, 1))
# num go terms
numGoTerms = apply(!is.na(xpvals), 2, sum)
# median of the actual scores
medianPval = apply(xpvals, 2, median, na.rm=T)
par(mar=margins)
barplot(medianPval, angle=45, density=50, main="Median p-value of GO terms", col=c(rainbow(n1),
    gray.colors(n2)), cex.names=textcex, las=las, space=barplot_space)
par(mar=margins)
numVerySignificantGOs = apply(xpvals<0.001, 2, sum, na.rm=T)
barplot(numVerySignificantGOs, angle=45, density=50, main="Number of GO terms with p < 0.001",
    col=c(rainbow(n1), gray.colors(n2)), cex.names=textcex, las=las, space=barplot_space)

# get the precentage of GOs with pval < 0.001
totalNumGOs = apply(!is.na(xpvals), 2, sum)
percentVerySignificantGOs = numVerySignificantGOs / totalNumGOs
percentVerySignificantGOs[which(is.na(percentVerySignificantGOs))]=0
par(mar=margins)
barplot(percentVerySignificantGOs, angle=45, density=80, main="Percentage of GO terms with p <
    0.001", col=c(rainbow(n1), gray.colors(n2)), cex.names=textcex, las=las,
    space=barplot_space, cex.main=1.7)

par(mar=margins)
names(numVerySignificantGOs) = gsub(names(numVerySignificantGOs), pattern='Unsup:',
    replace='Ensemble:')
barplot(numVerySignificantGOs, angle=45, density=80, main="Number of GO terms with p < 0.001",
    col=c(rainbow(n1), gray.colors(n2)), cex.names=1.5, las=las, space=barplot_space,
    cex.main=1.7)
par(mar=margins)
names(percentVerySignificantGOs) = gsub(names(percentVerySignificantGOs), pattern='Unsup:',
    replace='Ensemble:')
barplot(percentVerySignificantGOs, angle=45, density=80, main="Percentage of GO terms with p <
    0.001", col=c(rainbow(n1), gray.colors(n2)), cex.names=1.5, las=las, space=barplot_space,
    cex.main=1.7)

########################### Display item: rank the methods by the validations and statistics
    ########################################
# take the k=1, 2, 3, 4, 5

# Gene coverage and NGPG
files = names(flows_as_mats)
splitted_flows = which(grepl(files, pattern = "_(BP)|(CC)|(MF).txt", perl=T, ignore.case=T))
unsplitted_flow_files = files[-splitted_flows]
unsplitted_flows = flows_as_mats[unsplitted_flow_files]
names(unsplitted_flows) = getFileNameToToolName(unsplitted_flow_files)
all_flows = c(unsplitted_flows, unsupervised_k2ensemble_as_mats)
flow_genes = sapply(all_flows, getFlowGenes)
flow_gos = sapply(all_flows, getFlowGOs)
flow2numgenes = sapply(flow_genes, length)
flow2numannots = sapply(all_flows, length)/2
flow2numGOsPerGene = flow2numannots/flow2numgenes
coverage_rank = (length(flow2numgenes )-rank(flow2numgenes, "random")+1)
ngpg_rank = rank(flow2numGOsPerGene, "random")
stats_rank = rank(coverage_rank+ngpg_rank)
names(stats_rank) = gsub(names(stats_rank), pattern='Unsup:', replace='Ensemble:')

# Gold standard validation
mf1 = mf_gs[1, ];mf2 = mf_gs[2, ]
bp=colMeans(bp_gs)
mf1 = mf1[-which(grepl(names(mf1), pattern="Ensemble:k=6|7|8|9|(10)", perl=T))]
mf2 = mf2[-which(grepl(names(mf2), pattern="Ensemble:k=6|7|8|9|(10)", perl=T))]
bp = bp[-which(grepl(names(bp), pattern="Ensemble:k=6|7|8|9|(10)", perl=T))]
```

```
gs_r2 = (length(mf2)-rank(mf2, "random")+1)
gs_r3 = (length(mf1)-rank(mf1, "random")+1)
gs_r4 = (length(bp)-rank(bp, "random")+1)
gs_r = rank(gs_r3+gs_r4+gs_r2)

# Gene expression validation
numGood = numVerySignificantGOs;percentGood = percentVerySignificantGOs
numGood = numGood [-which(grepl(names(numGood ), pattern="Ensemble:k=6|7|8|9|(10)", perl=T))]
percentGood = percentGood[-which(grepl(names(percentGood), pattern="Ensemble:k=6|7|8|9|(10)",
    perl=T))]
ge_r2 = (length(numGood)-rank(numGood, "random")+1)
ge_r3 = (length(percentGood)-rank(percentGood, "random")+1)
ge_r = rank(ge_r2+ge_r3)

# Plot
all_names = union(names(ge_r), names(gs_r))
inters_names = intersect(names(ge_r), names(gs_r))
final_ranks = (ge_r[inters_names]+gs_r[inters_names]+stats_rank[inters_names])/3
cols = c(rep("black", 3), rep("white", length(final_ranks)-3))
par(mar=margins)
names(final_ranks) = gsub(names(final_ranks), pattern='Unsup:', replace='Ensemble:')
barplot(sort(final_ranks), ylab="Aggregated rank", col = cols, main="Ranking-based consolidation
    of the validations", cex.names=textcex, las=las)
##################################################
#################### QA ########################
#load("unsupervised_k2ensemble_as_mats.RData")
#load("C:/Users/dd/Desktop/hackathon/sample_data/gs/Sanjeev_gold_ITAG")
#preds = unsupervised_k2ensemble_as_hashes[[2]]
#gs = gs_as_hashes[["C:/Users/dd/Desktop/hackathon/sample_data/gs/Sanjeev_gold_ITAG.txt"]]
#sims = bp_sims_mat
```

MAIN.R

```
# IMPORTANT: Read the comments below on how to set the needed directories and files

# Set the working directory
WD = ""
setwd(WD)

# install required packages
#source("http://bioconductor.org/biocLite.R")
#biocLite("GOSemSim", destdir=".", lib=".", dep=T)
#biocLite("GO.db", destdir=".", lib=".", dep=T)
#install.packages("corrplot", destdir=".", lib=".", dep=T)
#install.packages("hash", destdir=".", lib=".", dep=T)
#install.packages("lattice", destdir=".", lib=".", dep=T)
#install.packages("multicore", destdir=".", lib=".", dep=T)

# load packages
#library(GOSemSim);library(hash);library(corrplot);library(GO.db)
# if the packages were installed locally
library(hash, lib='.');library(corrplot, lib='.');library(GO.db);library(multicore, lib='.')
#library(hash);library(corrplot);library(GO.db)

# load project functions;
# scripts_dir is the directory with the 'flow_comp_methods.R' script
scripts_dir = ""
source(paste(scripts_dir, "flow_comp_methods.R", sep="/"))

# IMPORTANT: the getGO2AnnotationType method uses both GO.db and a file we downloaded from the
    GO website (go2ont.txt).
# The go2ont file is given together with the projects file.
# Set the path to this directory
# This is an example:
additional_goinfo_file = "go2ont.txt"

# Set the input of the analysis.
# This file contains the output of the pipelines, the gold standard, and the gene expression data
```

```
# Thus, it should have 3 sub-directories (each contains required data explained below):
#     "flows" - the output of the pipelines
#     "gs - the gold standard file
#     "ge_data" - the gene expression matrices
# This is an example:
data_dir = "/home/gaga/davidama/hackathon/itag_data"

print (data_dir)
flow_res_dir = paste(data_dir, "flows", sep="/")
gold_standards_dir = paste(data_dir, "gs", sep="/")
gene_expression_dir = paste(data_dir, "ge_data", sep="/")
output_objects_dir = paste(data_dir, "output_objects", sep="/")
dir.create(output_objects_dir)

# in case the user wants to reread previously generated objects
#for (robj in dir(output_objects_dir)){
#     load(paste(output_objects_dir, robj, sep="/"))
#}

# read the output of the flows and restore in convinient data structures
files = paste(flow_res_dir, dir(flow_res_dir), sep="/")
# for windows
flow_preds = sapply(files, readFlowOutput)
flow_genes = sapply(files, getValueSetInColumn, column=1)
flow_GOs = sapply(files, getValueSetInColumn, column=2)
flows_as_mats = sapply(files, readFlowOutputTable)
flows_as_hashes = sapply(flows_as_mats, getgene2GOHash)

# remove ancestors of childrens from the annotations
# don't run this if there are no duplications in the data
# this will run for a long time
bplist=as.list(GOBPANCESTOR);mflist=as.list(GOMFANCESTOR);cclist=as.list(GOCCANCESTOR)
flows_after_removal = lapply(flows_as_hashes, removeDuplicationsFromAnnotations, bplist, cclist,
    mflist)
# unix version
#flows_after_removal = mclapply(flows_as_hashes, removeDuplicationsFromAnnotations, bplist,
    cclist, mflist, mc.cores=numCores)
#names(flows_after_removal) = names(flows_as_hashes)
flows_as_mats = list();flows_as_hashes=list();flow2numremoved=c()
for (f in files){
      flows_as_mats[[f]] = flows_after_removal[[f]][[2]]
      flows_as_hashes[[f]] = flows_after_removal[[f]][[1]]
      flow2numremoved[f] = flows_after_removal[[f]][[3]]
}
save(flows_as_mats, file=paste(output_objects_dir, "flows_as_mats.RData", sep="/"))
save(flows_as_hashes, file=paste(output_objects_dir, "flows_as_hashes.RData", sep="/"))
save(flow2numremoved, file=paste(output_objects_dir, "flow2numremoved.RData", sep="/"))

# $$$$$ STEP1 $$$$$: get basic statistics
flow2numgenes = sapply(flow_genes, length)
flow2numgos = sapply(flow_GOs, length)
flow2numannots = sapply(flow_preds, length)
flow2numGOsPerGene = flow2numannots/flow2numgenes
save(flow2numGOsPerGene , file=paste(output_objects_dir, "flow2numGOsPerGene.RData", sep="/"))
save(flow2numgenes, file=paste(output_objects_dir, "flow2numgenes.RData", sep="/"))
save(flow2numannots, file=paste(output_objects_dir, "flow2numannots.RData", sep="/"))

# $$$$$ STEP2 $$$$$: split the flows by GO terms:
# for each flow create a file for CC, MF, and BP
# annotations. If no annotation exists for one of the types
# the file is not created.
go2ontologyType = getGO2AnnotationType(additional_goinfo_file)
onts = unique(go2ontologyType)
for (f in files){
      currt = flows_as_mats[[f]]
      gos = as.character(currt[, 2])
      go_types3 = go2ontologyType[gos]
      f_name = strsplit(split = '\\.', f, perl=T)[[1]][1]
      for (o in onts){
            curr_out_file = paste(f_name, "_", o, ".txt", sep="")
```

```
            curr_inds = which(go_types3==o)
            # make sure that a file will be generated only if there are annotations of the
                tested type
            if (length(curr_inds)>0){write.table(file=curr_out_file, currt[curr_inds, ],
                sep="\t", quote=F, row.names=F, col.names=F)}
      }
}
# reread the data and save
files = paste(flow_res_dir, dir(flow_res_dir), sep="/")
flows_as_mats = sapply(files, readFlowOutputTable)
flows_as_hashes = sapply(flows_as_mats, getgene2GOHash)
save(flows_as_mats, file=paste(output_objects_dir, "flows_as_mats.RData", sep="/"))
save(flows_as_hashes, file=paste(output_objects_dir, "flows_as_hashes.RData", sep="/"))
flow_preds = sapply(flows_as_mats, getFlowPreds)
save(flow_preds, file=paste(output_objects_dir, "flow_preds.RData", sep="/"))

# $$$$$ STEP3 $$$$$: compare flows: GO-free
jaccard_matrix_annots = getJaccardMatrix(flow_preds)
save(jaccard_matrix_annots, file=paste(output_objects_dir, "jaccard_matrix_annots.RData",
    sep="/"))
# TODO: need to correct this
#jaccard_matrix_gos = getJaccardMatrix(flow_GOs)
#save(jaccard_matrix_gos, file=paste(output_objects_dir, "jaccard_matrix_gos.RData", sep="/"))
#jaccard_matrix_genes = getJaccardMatrix(flow_genes)
#save(jaccard_matrix_genes, file=paste(output_objects_dir, "jaccard_matrix_genes.RData",
    sep="/"))

# $$$$$ STEP4 $$$$$: get the GO similairty matrices or load given matrices
# option 1: load
#load("/home/gaga/davidama/hackathon/scripts/bp_sims_mat.RData")
#load("/home/gaga/davidama/hackathon/scripts/mf_sims_mat.RData")
#load("/home/gaga/davidama/hackathon/scripts/cc_sims_mat.RData")
load("bp_sims_mat.RData")
load("mf_sims_mat.RData")
load("cc_sims_mat.RData")

# option 2: calculate
#goterms = getGOTerms("MF", files, path=flows_path)
#mf_sims_mat = getSimMat("MF", goterms)
#save(mf_sims_mat, file=paste(output_objects_dir, "mf_sims_mat.RData", sep="/"))
#goterms = getGOTerms("BP", files, path=flows_path)
#bp_sims_mat = getSimMat("BP", goterms)
#save(bp_sims_mat, file=paste(output_objects_dir, "bp_sims_mat.RData", sep="/"))
#goterms = getGOTerms("CC", files, path=flows_path)
#cc_sims_mat = getSimMat("CC", goterms)
#save(cc_sims_mat, file=paste(output_objects_dir, "cc_sims_mat.RData", sep="/"))

# $$$$$ STEP5 $$$$$: compare flows: GO-based
flow_go_sims = list()
for (o in onts){
      if (grepl(o, pattern="cc", ignore.case=T)){next}
      if (grepl(o, pattern="mf", ignore.case=T)){sims = mf_sims_mat}
      if (grepl(o, pattern="bp", ignore.case=T)){sims = bp_sims_mat}
      curr_files = files[which(grepl(files, pattern = o))]
      if (length(curr_files)==0){next}
      curr_go_terms = getGOTerms(o, curr_files, flow_mats=flows_as_mats)
      curr_sim_mat = getSemSimMatrix(flows_as_hashes[curr_files], sims=sims)
      flow_go_sims[[o]] = curr_sim_mat
}
save(flow_go_sims, file=paste(output_objects_dir, "flow_go_sims.RData", sep="/"))

# $$$$$ STEP6 $$$$$: compare flows to gold standard
gs_files = dir(gold_standards_dir)
gs_files = paste(gold_standards_dir, gs_files, sep="/")
gs_as_mats = sapply(gs_files, readFlowOutputTable)
gs_as_hashes = sapply(gs_as_mats, getgene2GOHash)
validation_gold_stand_go_free = getFlowsPerformance(flows_as_mats, getAccuracyMeasures,
    gs_files, gs_as_mats)
save(validation_gold_stand_go_free, file=paste(output_objects_dir,
    "validation_gold_stand_go_free.RData", sep="/"))
```

```
validation_gold_stand_go_bp = getFlowsPerformance(flows_as_hashes, getGOBasedAccuracyMeasures,
    gs_files, gs_as_hashes, sims=bp_sims_mat)
save(validation_gold_stand_go_bp, file=paste(output_objects_dir,
    "validation_gold_stand_go_bp.RData", sep="/"))
validation_gold_stand_go_mf = getFlowsPerformance(flows_as_hashes, getGOBasedAccuracyMeasures,
    gs_files, gs_as_hashes, sims=mf_sims_mat)
save(validation_gold_stand_go_mf, file=paste(output_objects_dir,
    "validation_gold_stand_go_mf.RData", sep="/"))

# $$$$$ STEP7 $$$$$:: Analyze gene expression data
goterms = getGOTerms("", files=names(flows_as_mats), flow_mats=flows_as_mats)
goterms = setdiff(goterms, "")
ge_data_files = paste(gene_expression_dir, dir(gene_expression_dir), sep="/")
max_go_term_size=500;min_go_term_size=3
curr_mats = ge_data_files
curr_bp_files = names(flows_as_mats)[which(grepl(names(flows_as_mats), pattern="_BP.txt"))]
ge_validation_results = run_gene_expression_validation(flows_as_mats[curr_bp_files], curr_mats,
    goterms, max_go_term_size=500, min_go_term_size=3)
flows_ge_validation_results_pvals = ge_validation_results[[1]]
flows_ge_validation_results_meancorr = ge_validation_results[[2]]
save(flows_ge_validation_results_pvals, file=paste(output_objects_dir,
    "flows_ge_validation_results_pvals.RData", sep="/"))
save(flows_ge_validation_results_meancorr, file=paste(output_objects_dir,
    "flows_ge_validation_results_meancorr.RData", sep="/"))

# $$$$$ STEP8 $$$$$:: Generate a union of all flows and rerun the comparisons
#################### Unsupervised ###########################
# Ensemble of BPs
curr_files = files[which(grepl(files, pattern="_BP"))]
# in the original analysis: remove metastudent and one of the mcls
bp_unsupervised_k2ensemble = getAllKEnsembleOfFlows(curr_files, flows_as_mats,
    as.list(GOBPANCESTOR))
save(bp_unsupervised_k2ensemble, file=paste(output_objects_dir,
    "bp_unsupervised_k2ensemble.RData", sep="/"))
# Ensemble of MFs
curr_files = files[which(grepl(files, pattern="_MF"))]
# in the original analysis: remove metastudent and one of the mcls
mf_unsupervised_k2ensemble = getAllKEnsembleOfFlows(curr_files, flows_as_mats,
    as.list(GOMFANCESTOR))
save(mf_unsupervised_k2ensemble, file=paste(output_objects_dir,
    "mf_unsupervised_k2ensemble.RData", sep="/"))
# Ensemble of CCs
curr_files = files[which(grepl(files, pattern="_CC"))]
# in the original analysis: remove metastudent and one of the mcls
cc_unsupervised_k2ensemble = getAllKEnsembleOfFlows(curr_files, flows_as_mats,
    as.list(GOCCANCESTOR))
save(cc_unsupervised_k2ensemble, file=paste(output_objects_dir,
    "cc_unsupervised_k2ensemble.RData", sep="/"))
# merge the ensembles
unsupervised_k2ensemble_as_mats = list()
Ks = sort(unique(c(names(bp_unsupervised_k2ensemble), names(mf_unsupervised_k2ensemble),
    names(mf_unsupervised_k2ensemble))))
for (k in Ks){
      curr_bp_mat = bp_unsupervised_k2ensemble[[k]]
      curr_mf_mat = mf_unsupervised_k2ensemble[[k]]
      curr_cc_mat = cc_unsupervised_k2ensemble[[k]]
      curr_mat = rbind(curr_bp_mat, curr_mf_mat, curr_cc_mat)
      unsupervised_k2ensemble_as_mats[[k]] = curr_mat
}
names(unsupervised_k2ensemble_as_mats) = paste("Unsup:k=",
    names(unsupervised_k2ensemble_as_mats), sep="")
save(unsupervised_k2ensemble_as_mats, file=paste(output_objects_dir,
    "unsupervised_k2ensemble_as_mats.RData", sep="/"))
unsupervised_k2ensemble_as_hashes = sapply(unsupervised_k2ensemble_as_mats, getgene2GOHash)
names(unsupervised_k2ensemble_as_hashes)=names(unsupervised_k2ensemble_as_mats)
save(unsupervised_k2ensemble_as_hashes, file=paste(output_objects_dir,
    "unsupervised_k2ensemble_as_hashes.RData", sep="/"))

# GS validation
```

```
unsupervised_validation_gold_stand_go_free =
    getFlowsPerformance(unsupervised_k2ensemble_as_mats, getAccuracyMeasures, gs_files,
    gs_as_mats)
save(unsupervised_validation_gold_stand_go_free, file=paste(output_objects_dir,
    "unsupervised_validation_gold_stand_go_free.RData", sep="/"))
unsupervised_validation_gold_stand_go_bp =
    getFlowsPerformance(unsupervised_k2ensemble_as_hashes, getGOBasedAccuracyMeasures, gs_files,
    gs_as_hashes, sims=bp_sims_mat)
save(unsupervised_validation_gold_stand_go_bp, file=paste(output_objects_dir,
    "unsupervised_validation_gold_stand_go_bp.RData", sep="/"))
unsupervised_validation_gold_stand_go_mf =
    getFlowsPerformance(unsupervised_k2ensemble_as_hashes, getGOBasedAccuracyMeasures, gs_files,
    gs_as_hashes, sims=mf_sims_mat)
save(unsupervised_validation_gold_stand_go_mf, file=paste(output_objects_dir,
    "unsupervised_validation_gold_stand_go_mf.RData", sep="/"))
# GE validation
goterms = getGOTerms("", files=names(unsupervised_k2ensemble_as_mats),
    flow_mats=unsupervised_k2ensemble_as_mats)
ge_data_files = paste(gene_expression_dir, dir(gene_expression_dir), sep="/")
max_go_term_size=500;min_go_term_size=3
curr_mats = ge_data_files
ge_validation_results = run_gene_expression_validation(unsupervised_k2ensemble_as_mats,
    curr_mats, goterms, max_go_term_size=500, min_go_term_size=3)
unsupervised_flows_ge_validation_results_pvals = ge_validation_results[[1]]
unsupervised_flows_ge_validation_results_meancorr = ge_validation_results[[2]]
save(unsupervised_flows_ge_validation_results_pvals, file=paste(output_objects_dir,
    "unsupervised_flows_ge_validation_results_pvals.RData", sep="/"))
save(unsupervised_flows_ge_validation_results_meancorr, file=paste(output_objects_dir,
    "unsupervised_flows_ge_validation_results_meancorr.RData", sep="/"))
```

Methods S6: The supervised ensemble

As an alternative approach to our original *unsupervised ensemble* method we considered the following approach. First, given a set of pipelines the basic step is to run the unsupervised ensemble with k=1. We denote this step as *simple merge.* The second step is supervised, and takes as input both the results of each pipeline and the gold standard annotations. These annotations are used to rank the pipelines by their average F-measure. Finally, the *supervised ensemble* method chooses the top ℓ pipelines and integrates them using the simple merge step.

Since the supervised approach uses the gold standard annotations as input, we compared the two ensemble methods only based on gene co-expression, as described in the main text (Figure 6). The comparison using the potato PGSC data is shown in the figure below.

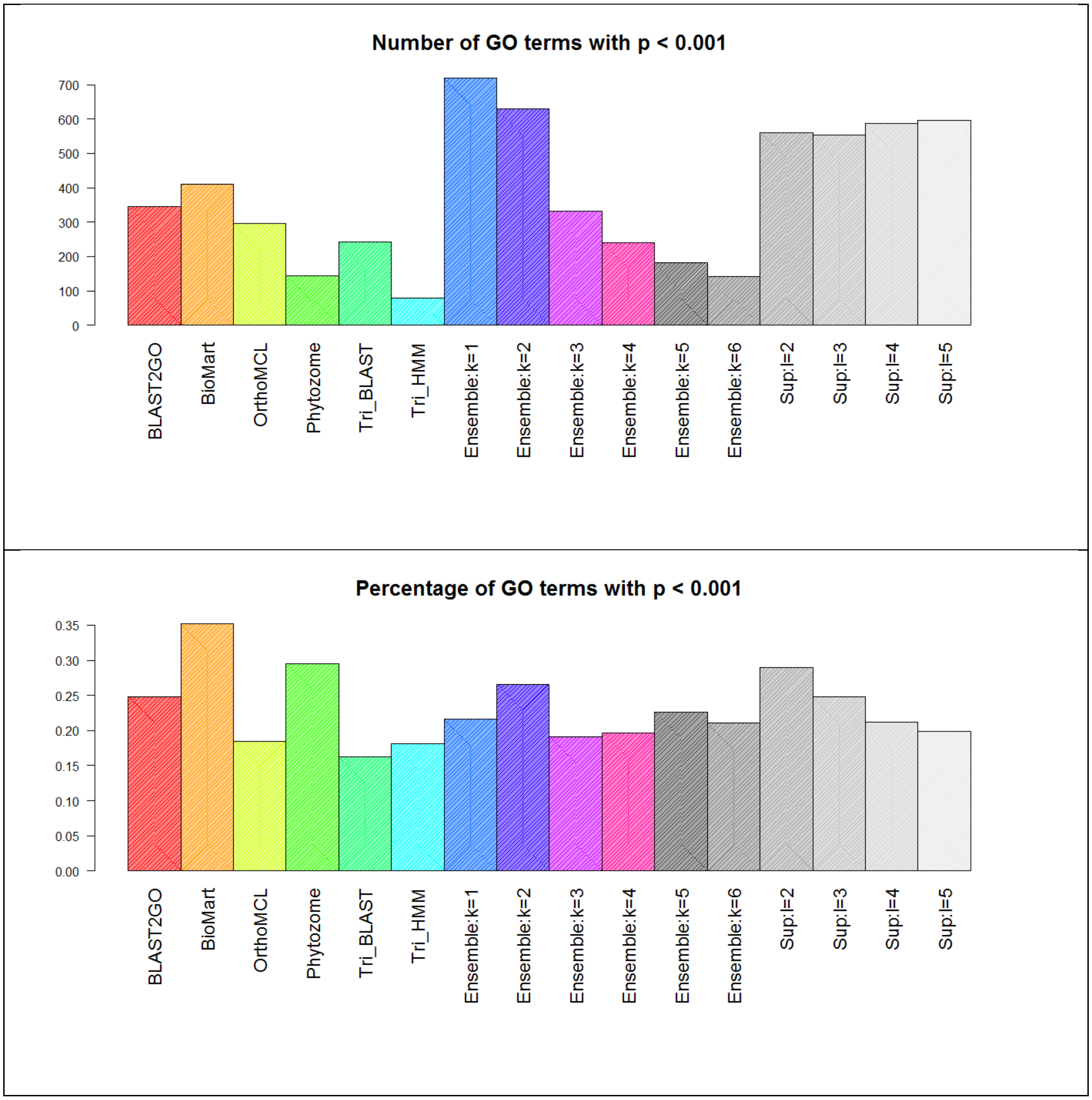

The results show that for the potato data, the two ensemble approaches provide comparable results. The original unsupervised ensemble (named *Ensemble:k* in the figure above, as in the main text) has superior results with k=1 or k=2 in terms of the number of highly co-expressed GO terms. However, with ℓ=2, the supervised ensemble has slight advantage over the unsupervised ensemble (k=2) in terms of the percentage of highly co-expressed GO terms.

Based on the comparison above, we conclude that for the currently available potato data, the two approaches have similar performance. As the unsupervised ensemble is simpler and uses less information, we chose to use it for annotating the potato genes. The supervised ensemble implementation is also available as part of the R code, and can be utilized in future studies.

<u>Methods S7: Co-expression of the gold standard genes</u>

The figure below shows histograms of the co-expression values between all gene pairs, and between gene pairs in the gold standard (GS) set. The distribution of the GS genes has slightly higher mean (0.007 vs. 0.014) but both scores are very close to zero, and the distributions are highly overlapping.

The genes in the gold standard fall into 17 different GO process categories. The average co-expression of these categories is not higher than that of for random gene sets of the same size.

Both tests suggest that the co-expression information is complementary to the set of GS genes and is not biasing the co-expression analysis.

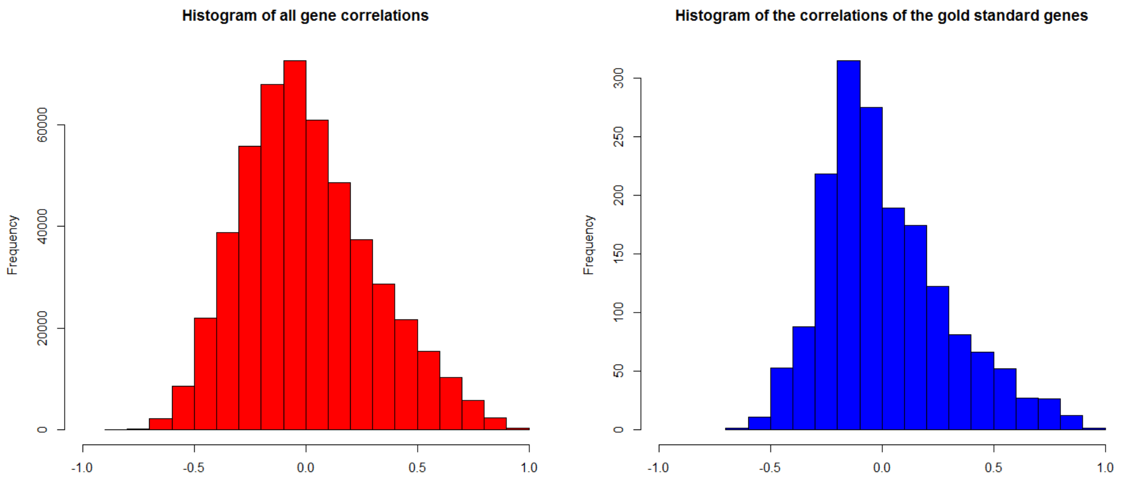

Supplement: Additional file 1: Figure S1. — ITAG pipeline similarity, Figure S2. ITAG gold standard validation, Figure S3. ITAG gene expression validation and Methods S1-5. [file 12870_2014_329_MOESM1_ESM.pdf]
